# Optimizing Infrazygomatic Miniscrew Insertion Parameters: Systematic Review and Meta-Regression Analysis of Bone Thickness by Insertion Height, Angulation, and Anatomical Position

**DOI:** 10.3390/jcm14114005

**Published:** 2025-06-05

**Authors:** Kais Hijazi Muwaquet, Susana Muwaquet Rodriguez, Marcela Ferrer Molina, Tawfiq Hijazi Alsadi

**Affiliations:** 1 Department of Dentistry, Universidad Católica de Valencia, 46001 Valencia, Spain; kais.hijazi@mail.ucv.es; 2 Department of Restorative Dentistry and Endodontics, Faculty of Medicine and Health Science, Catholic University of Valencia (UCV), C/Quevedo, 2, 46001 Valencia, Spain; susana.muwaquet@ucv.es; 3 Department of Orthodontics, Faculty of Medicine and Health Science, Catholic University of Valencia, 46001 Valencia, Spain; marcela.ferrer@ucv.es

**Keywords:** bone thickness, CBCT, facial pattern, infrazygomatic miniscrews, insertion angle, insertion height, zygomatic crest

## Abstract

**Introduction:** Infrazygomatic crest (IZC) miniscrews are widely used for skeletal anchorage in orthodontics. Despite their growing popularity, the optimal insertion parameters—such as height, angulation, and anatomical position—remain controversial, with existing studies offering inconsistent and fragmented data. **Aim:** To determine the optimal insertion position, height, and angulation of infrazygomatic miniscrews to maximize bone insertion using cone-beam computed tomography (CBCT) analysis and to investigate the influence of facial skeletal patterns on IZC bone morphology. **Methods:** This review was conducted according to the PRISMA 2020 guidelines. A comprehensive electronic search was performed across six databases: PubMed, Scopus, Web of Science, Cochrane, EBSCO, and Google Scholar. Studies reporting CBCT-based IZC bone thickness were included. A meta-analysis was conducted using a random-effects model, and meta-regression was applied to assess the relationship between insertion height, angulation, and bone thickness. The STROBE checklist was used to assess the quality of the included observational studies. **Results:** Seventeen studies comprising a total of 1840 CBCT-based measurements were included. The meta-regression revealed a significant inverse relationship between insertion height and bone thickness (β = −0.53; *p* < 0.001) and a positive correlation with angulation (β = 0.09; *p* < 0.001). The U67 region refers to the anatomical area between the maxillary first and second molars, adjacent to the infrazygomatic crest and zygomatic buttress, which with an insertion height of 9.9 mm and 80° angulation, demonstrated the highest mean cortical bone thickness (3.52 mm). There was no evidence of a significant association between facial pattern and bone thickness (*p* = 0.878). **Conclusions:** This review presents the first predictive model for IZC miniscrew placement based on meta-regression. The findings support the U67 site at 9.9 mm height and 80° angulation as the optimal insertion protocol. These data-driven guidelines provide clinicians with practical, evidence-based direction for improving miniscrew stability and minimizing complications.

## 1. Introduction

The basic principle behind orthodontic tooth movement relies on Newton’s third law, which states that every force will produce an equal reaction. The fundamental principle of orthodontics requires anchorage management to stop unwanted movements from occurring in reactive units. The current anchorage control techniques, such as transpalatal arches and Nance buttons, together with headgear and other intraoral and extraoral appliances, have produced varied results, but their effectiveness remains limited by patient compliance and anatomical restrictions [1]. Temporary anchorage devices (TADs) have transformed clinical orthodontics through skeletal anchorage, which provides a dependable alternative to anchorage that does not rely on patient compliance [2,3]. Miniscrews represent one of the TAD types that have become prominent because they provide easy placement and cost-effective absolute anchorage without requiring osseointegration [2].

**Figure 1:**
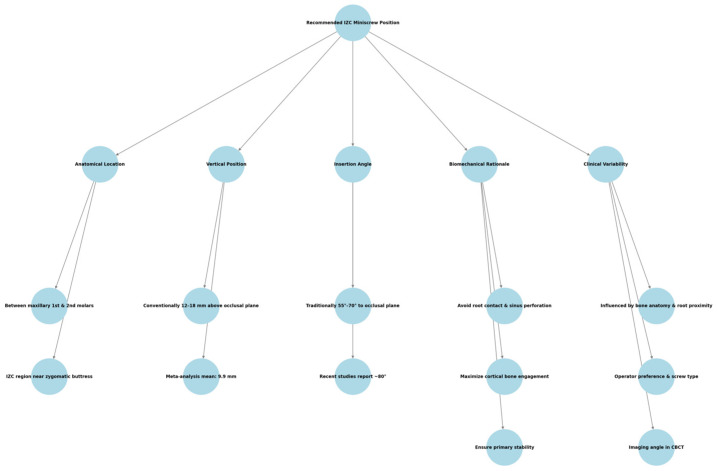
A schematic representation of the anatomical site and recommended infrazygomatic crest (IZC) miniscrew parameters.

The infrazygomatic crest (IZC) stands out as the best placement location for maxillary miniscrews. The IZC position between the maxillary alveolar crest and the zygomatic process offers a non-dentoalveolar area with high bone density and sufficient cortical thickness while minimizing root interference, thus making it suitable for extra-alveolar TAD placement [4,5,6]. IZC miniscrews receive benefits from their location in dense cortical bone, which produces better primary stability together with a wider insertion zone compared to inter-radicular miniscrews that face restrictions from root proximity and thin alveolar bone [7,8]. The recommended position for infrazygomatic crest (IZC) miniscrew insertion is in the buccal cortical bone located between the maxillary first and second molars, in the region of the zygomatic buttress. The vertical height is generally described as being between 12 and 18 mm above the occlusal plane, with insertion angles typically ranging from 55° to 70° relative to the occlusal or horizontal plane. These values aim to optimize cortical bone engagement while avoiding root proximity and sinus penetration. However, variations exist across the literature, and recent studies—including those analyzed in this review—have reported slightly lower vertical heights and steeper insertion angles, reflecting the evolving clinical practice. (Figure 1) provides a schematic representation of the anatomical site and commonly recommended parameters [9,10].

**Figure 2:**
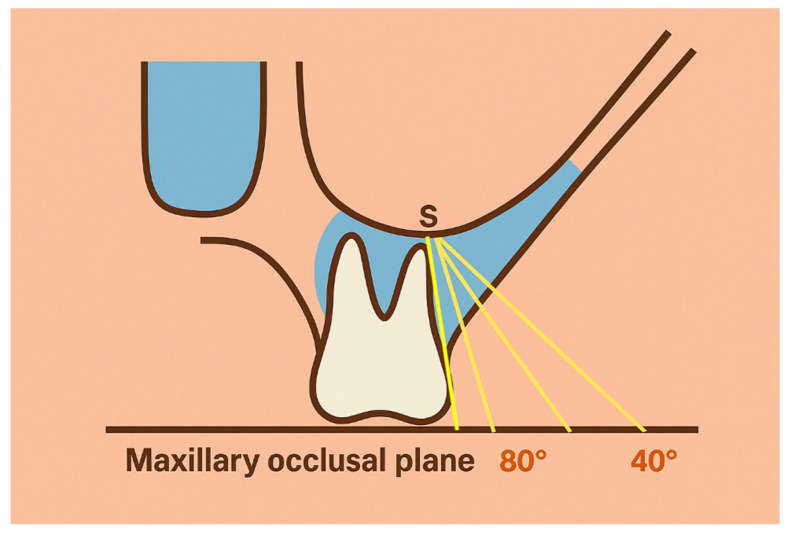
Diagram illustrating reference lines and points in relation to the maxillary first molar and Zygomatic Buttress (U67).

Liou et al. pointed out that some reference lines and points were set on chosen coronal slices to quantify the lateral wall thickness of the maxillary sinus and the thickness of the infrazygomatic (IZ) crest. The first reference line was the maxillary occlusal plane, which was defined as the plane passing through the mesiobuccal cusps of both maxillary first molars. The second reference line was a tangent line to the buccal surface of the mesiobuccal root of the maxillary first molar. The point where this tangent line crossed the floor of the maxillary sinus was called the sinus point (S point) and was used as a significant landmark for the measurements. From point S, additional reference lines were drawn at 5° intervals to the maxillary occlusal plane. These lines also indicated the angles at which miniscrews were to be placed. The points at which these reference lines crossed the lateral surface of the IZ crest were selected (Figure 2) [11]. When anatomical reference lines were specified, we adopted the mesiobuccal (MB) root of the maxillary first molar as the standard reference point, for consistency and comparability with the most cited anatomical models.

Medical practitioners utilize IZC miniscrews to perform different complicated orthodontic interventions, including anterior en masse retraction and maxillary arch distalization and posterior intrusion and full-arch intrusion for Class II malocclusions with vertical maxillary excess and skeletal asymmetries [7,12,13]. The placement of these anchors enables unblocked tooth movement in multiple directions, which produces results that traditional anchorage systems cannot achieve. Research evidence shows IZC anchorage helps decrease vertical dimensional changes and control occlusal plane rotation during distalization mechanics [14].

Miniscrews placed in the IZC area create mechanical interlocking that does not require osseointegration because precise planning is essential to achieve primary stability while preventing complications such as sinus perforation or screw mobility [2,15]. The success of IZC miniscrews primarily depends on bone density and cortical thickness, and CBCT-based studies confirm that the IZC’s bone quality exceeds that of other intraoral locations [1,6,16]. The success stability of implants and their failure risks depend on the patient’s age and vertical skeletal pattern and the screw dimensions, as well as the insertion angle [4,17,18]. Patients with elevated mandibular plane angles show thinner cortical bone, which might reduce miniscrew success, but research in this field shows conflicting results [3,4,7].

The high success rate of IZC miniscrews reaches 90% to 95%, but surgical complications still happen. These include gingival overgrowth, mucosal irritation, and the rare occurrences of miniscrew loosening or sinus involvement, which tend to happen when screws are inserted too deeply or at improper angles [15,19,20]. A small sinus perforation of 2 mm or less is considered clinically acceptable and it does not affect success rates, especially when using stainless steel screws with a 2 mm diameter [18]. The essential use of CBCT imaging techniques remains critical for both preoperative bone volume assessment and accident prevention [21].

The versatility of IZC miniscrews in clinical practice, combined with their biomechanical benefits, makes them the preferred option for complex orthodontic anchorage needs. Research shows that experts fail to agree on the most effective insertion parameters, including height and orientation, along with anterior–posterior placement for maximizing stability and decreasing failure incidence. Anatomical, patient-specific differences in craniofacial structure, together with skeletal characteristics, affect IZC bone density and thickness, which complicates the development of standardized placement protocols [1,3,5,7]. While prior studies have explored bone thickness at various IZC locations, this meta-analysis is the first to quantify the combined impact of insertion height, angulation, and position using meta-regression modeling, synthesizing findings from CBCT-based studies and clinical trials in order to provide evidence-based guidelines to clinicians for the optimal placement of IZC miniscrews to minimize the likelihood of complications and improve the predictability and efficiency of orthodontic treatment outcomes.

## 2. Hypothesis

The research depends on the idea that insertion height together with angulation and mesio-distal positioning serve as quantifiable factors for determining IZC miniscrew placement bone availability.

**H0.**  *Null hypothesis: The placement position and height and angle of infrazygomatic miniscrews do not produce substantial effects on the maximum insertion bone area. The null hypothesis also establishes that facial skeletal patterns do not correlate with bone thickness measurements.*

**H1.**  *Alternative hypothesis: Establishes two distinct claims: the position, height, and angle of placement of the infrazygomatic miniscrews have significant effects on the maximum amount of bone insertion, and the facial growth pattern significantly affects the anatomical characteristics of the IZC area.*

## 3. Objectives

This research aims to analyze the effects of insertion location and height and angle on bone thickness throughout the infrazygomatic crest area using CBCT study data. The research uses a meta-analysis to identify the optimal anatomical placement parameters for IZC miniscrews to maximize cortical engagement and lead to better clinical results.

More specifically, this review seeks to identify if infrazygomatic bone thickness differs between people with unique facial skeletal patterns and how these differences affect miniscrew placement success rates in separate subgroups.

## 4. Materials and Methods

### 4.1. Study Design

This research followed the Preferred Reporting Items for Systematic Reviews and Meta-Analyses (PRISMA) guidelines to perform a systematic review and meta-analysis while using a predetermined protocol for methodological transparency and reproducibility [22], and it does not need registration. The review assessed how bone thickness in the infrazygomatic crest (IZC) region relates to insertion parameters, including height, angulation, and mesio-distal positioning, through the analysis of CBCT imaging data from observational studies. The assessment of the influence of facial skeletal patterns on bone thickness occurred when relevant data were present. The following structure was used to formulate the PICO question for the review:

P (Population): Patients receiving infrazygomatic miniscrew treatment.

I (Intervention): The measurement of bone thickness in various IZC regions.

C (Comparison): Different insertion heights, angles, and positions.

O (Outcome): Maximum bone thickness available for miniscrew insertion.

S (Statistical Analysis): Meta-regression to explore associations between parameters and bone thickness.

Thus, the PICO question was as follows: “Do different insertion positions, heights and angulations contribute in the measurement of bone thickness in different infrazygomatic crest areas in order to obtain the maximum bone thickness for the best possible miniscrew insertion in patients intending to receive infrazygomatic miniscrew treatments?”.

### 4.2. Eligibility Criteria

The eligibility criteria for the studies included adherence to the following conditions, stated below in (Table 1 and Table 2).

### 4.3. Search Strategy and Study Selection

The research included a complete electronic literature search of six databases, including PubMed, EBSCOhost, Scopus, Web of Science, Cochrane Library, and Google Scholar. Researchers used Boolean operators and MeSH terms, which included “infrazygomatic” together with “zygomatic crest” and “miniscrew” and “insertion angle” and “bone thickness” and “CBCT”. Additionally, the Boolean operators “AND” or “OR” were used alongside the formulated PICO question. The initial search took place in January 2025, followed by a repeat search in February 2025 to retrieve newly added articles (Table 3).

The search outcomes were transferred to a citation management tool to remove duplicate entries. Three stages comprised the selection process, which started with a title screening followed by an abstract review and ended with a full-text assessment. The reviewers settled their disagreements through group discussion until they reached an agreement. To calculate the inter-examiner agreement, the Cohen’s Kappa value was calculated, thus obtaining a *k*-value = 0.841; this falls in the range of 0.82 < κ < 1.00, consequently classifying the agreement between the examiners as “almost perfect”.

### 4.4. Data Extraction

A systematic data extraction process started after the completion of article selection. The relevant information from each study was documented in a master table, which contained the article title, author(s), year of publication, total sample size, study design, and facial skeletal pattern classification information, when available. Each study received an evaluation to determine whether it included mean bone thickness measurements at different insertion locations, together with insertion height and angle and mesio-distal position information (Table 4 and Table 5).

Numerical data, including standard deviations and *p*-values, along with insertion height ranges, were extracted from available studies for use in the meta-regression analysis. The standardization process was applied to relative insertion height measurements by using anatomical reference points (e.g., 7.5 mm from CEJ to occlusal plane) that were derived from anatomical studies [23].

In cases where the insertion height was measured from the alveolar crest between the mesial and distal alveolar bone crest of the maxillary first molars, the value 8.01 mm was also used as a reference [24]. This value offers precise anatomical data that can be used for standardization and proper integration into meta-analytical models while maintaining data consistency throughout the sample set. This structured approach helped in ensuring that only systematic and thorough data relevant to the review was collected.

### 4.5. Data Synthesis

Researchers have conducted an exhaustive systematic review of the literature, turning out a final selection of 17 articles [1,5,10,11,25,26,27,28,29,30,31,32,33,34,35,36,37] reporting information about the bone thickness measured for different combinations of height, angulation, and positions on CBCT images.

- Heights ranged from 9.9 to 19.01 mm.
- Angles ranged from 40 up to 80° (steps of 5°).
- Antero-posterior positions comprised 4 options: U6M (the mesiobuccal root of upper first molar), U6D (the distobuccal root of upper first molar), U67 (between the upper first and second molars), and U7M (the mesiobuccal root of upper second molar).

Simple mixed-effects models (meta-regression) for each moderator variable height, angle, and position were estimated to explain the primary outcome (bone thickness). Non-adjusted beta coefficients were estimated with corresponding Z statistics, 95% confidence intervals, and *p*-values for nullity tests.

A restricted maximum-likelihood estimator was used in the model. The I2 index was also calculated, representing the amount of between-studies variability compared to the total variability. The R2 was reported as an indicator of the amount of between-studies heterogeneity explained by each predictor. Then, a multiple model was estimated including all the relevant parameters, obtaining adjusted beta coefficients. The effect of the facial pattern was assessed in the subsample of articles providing this information. The level of significance used in the analysis was 5% (αlfa = 0.05). The software used was R 4.3.1 (R Core Team (2018). R: a language and environment for statistical computing. R Foundation for Statistical Computing, Vienna, Austria (http://www.R-project.org/). Where possible, facial pattern subgroups (brachyfacial, mesofacial, dolichofacial) were analyzed, and multivariate models were generated to control for potential covariate effects.

### 4.6. Quality and Risk of Bias Assessment

Two instruments were used to assess the studies’ quality and minimize any selection bias: the STROBE checklist was applied to cross-sectional, observational studies to assess the reporting of sample selection, methodology, and statistical procedures [38]. The PRISMA checklist was used to ensure that the systematic review and meta-analysis components were reported in detail. Each included study was independently rated and classified according to its methodological robustness. Any discrepancies in scoring were resolved by a consensus [38]. (both tables added in Supplementary Files).

## 5. Results

### 5.1. Selection of Studies: PRISMA Flowchart

**Figure 3:**
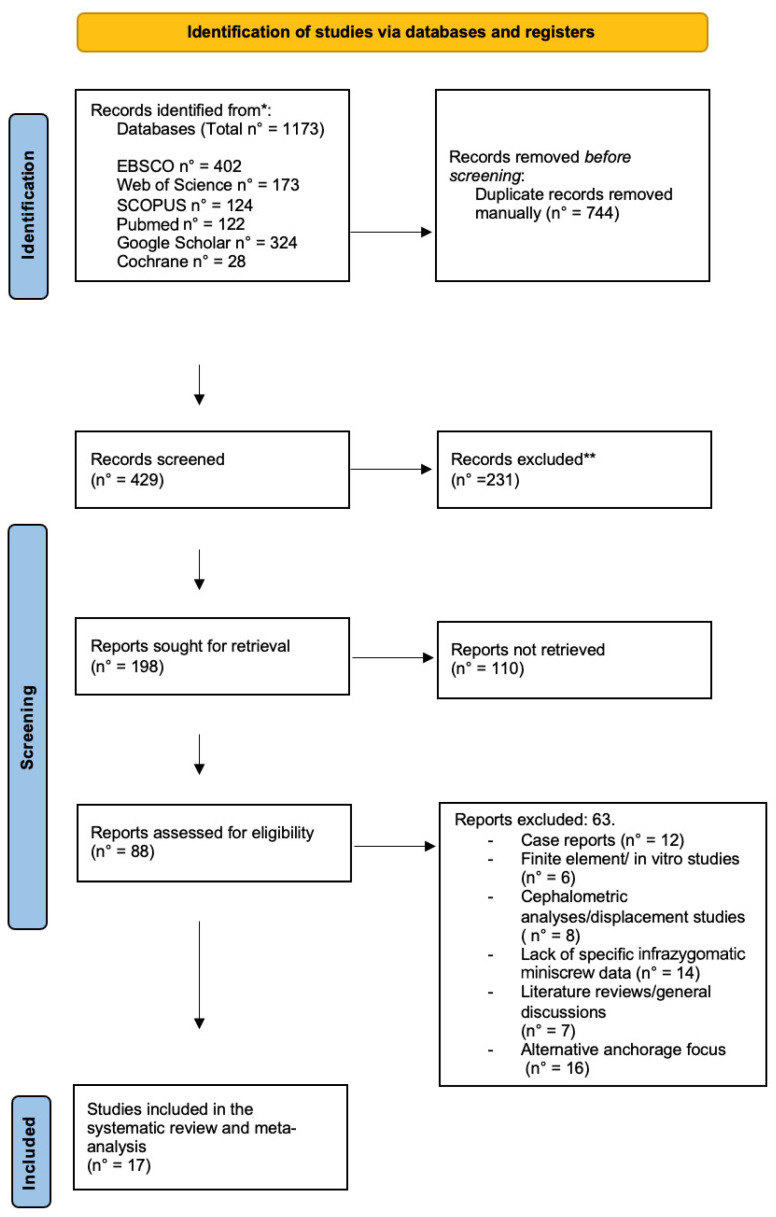
Prisma flow chart demonstrating the scheme that was followed in the selection of articles. * *p* < 0.05; ** *p* < 0.01.

After searching through the databases, a total of 1173 articles were found, as follows: EBSCO (*n* = 402), Google Scholar (*n* = 324), Web of Science (*n* = 173), SCOPUS (*n* = 124), Pubmed (*n* = 122), and Cochrane (*n* = 28). Secondly, 744 records were removed from the list before screening, and thereafter, 429 articles were left for review. Upon examining the titles and abstracts of these articles, 231 of them were excluded as they were unrelated to our research focus. Next, 198 reports were sought for retrieval, of which, 110 could not be retrieved. An exhaustive evaluation of eligibility was carried out on the 88 articles that remained. During the assessment of the study materials, 63 articles were found not to meet the inclusion criteria for several reasons. These reasons included twelve case reports; six finite element or laboratory studies; eight articles centered on cephalometric analyses or displacement patterns; fourteen articles with a lack of information on miniscrews; seven literature reviews and general discussions; and sixteen studies focusing on different anchorage sites, like the mandibular buccal shelf, zygoma plates, and cervical vertebrae. After selecting studies through a process of evaluation and scrutiny, 17 studies that met our specific criteria were identified [1,5,10,11,25,26,27,28,29,30,31,32,33,34,35,36,37] and were included in the comprehensive meta-analysis. This process is outlined below (Figure 3).

### 5.2. Effect of Height

**Figure 4:**
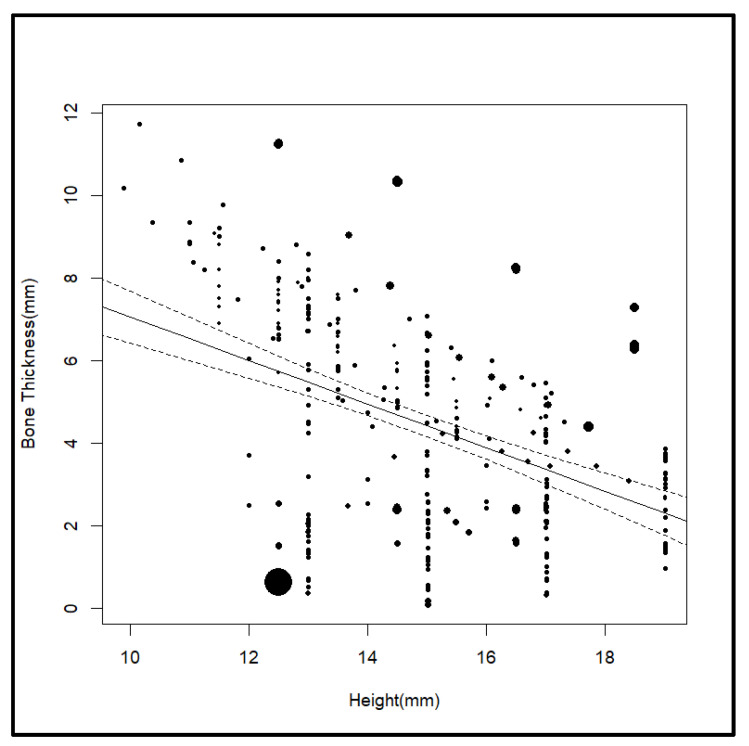
Effect of height.

The first meta-regression analysis was performed to evaluate the effect of height on bone thickness in the infra zygomatic crest region without controlling for other variables. The results showed a statistically significant negative correlation between height and bone thickness (β = −0.53, *p* < 0.001), meaning that for every 1 mm increase in height, bone thickness decreased by an average of 0.53 mm. The intercept of the model was also significant (β = 12.3, *p* < 0.001), indicating that the baseline thickness was not equal to zero. Although height alone explained 19.2% of the variance in bone thickness (R^2^ = 19.2%), a large proportion of heterogeneity (80.8%) remained unexplained, with the test for residual heterogeneity also being significant (*p* < 0.001). These findings indicate that although height is an important predictor, other unmeasured factors are likely to contribute to the variability in bone thickness. Clinically, this inverse relationship means that higher insertion points, which are typically found in taller individuals, may have thinner bone, which may affect the placement and stability of orthodontic miniscrews. Further studies that include other covariates, such as age, sex, and craniofacial morphology, are suggested to improve the understanding of the factors that affect bone thickness in this region (Table 6) (Figure 4).

### 5.3. Effect of Angulation

**Figure 5:**
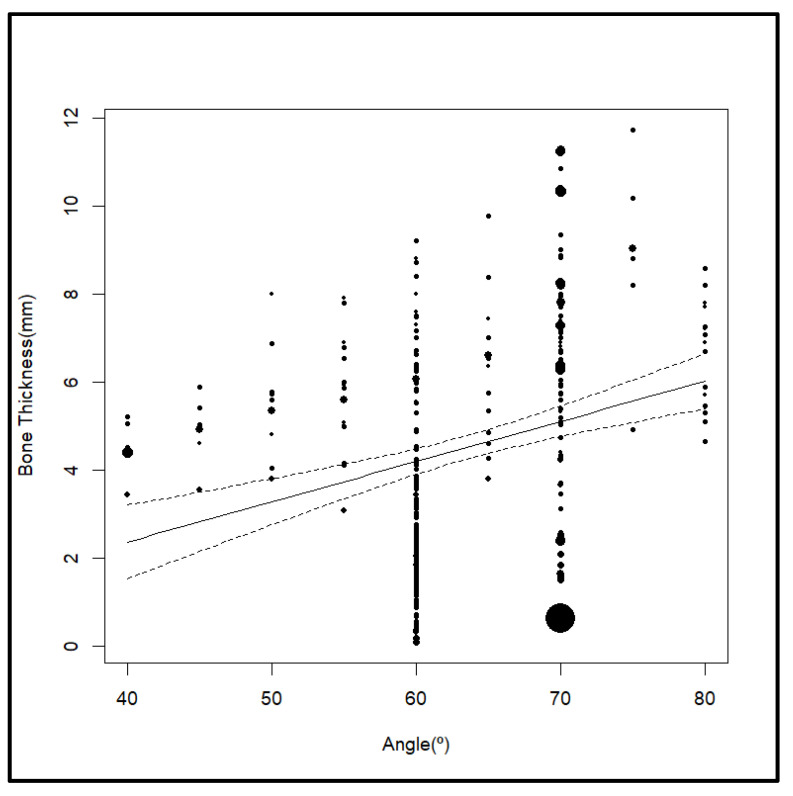
Effect of angulation.

Angulation was significantly correlated with bone thickness (*p* < 0.001). For each additional 1° of angle, bone thickness increased on average by +0.09 mm (Table 7) (Figure 5).

The overall heterogeneity between studies was very high (I^2^ = 99.9%), but (R^2^ =) 7.54% of this heterogeneity could be explained by angle. Notice that it is a smaller amount than the one obtained for height, although it is significant too. The residual heterogeneity was estimated at 92.5% (*p* < 0.001).

### 5.4. Effect of Position

A meta-regression analysis was performed to assess the effect of miniscrew insertion position on bone thickness, with U6M as the reference group. The results indicated that position was significantly associated with bone thickness (*p* < 0.001), and the U67 position had a statistically significant increase in bone thickness compared to U6M (β = 1.51, *p* < 0.001), meaning an average increase of 1.51 mm. U6D (β = −0.36, *p* = 0.337) and U7M (β = −0.46, *p* = 0.319) did not differ significantly from U6M. When the reference group was changed in complementary models, U67 was always found to have a greater bone thickness than all the other positions. The model explained 10.4% of the total heterogeneity (R^2^ = 10.4%), and a large portion of it (89.6%) remained as residual heterogeneity (*p* < 0.001), indicating the presence of other unmeasured variables. Forest plots showing all 331 subgroups showed that U67 positions were always located to the right, meaning higher bone thickness. A subsequent analysis that averaged the subgroups by position within each study also confirmed U67 as a distinct group with a higher bone thickness and narrower confidence intervals, which supports the robustness of this finding (Table 8).

**Figure 6:**
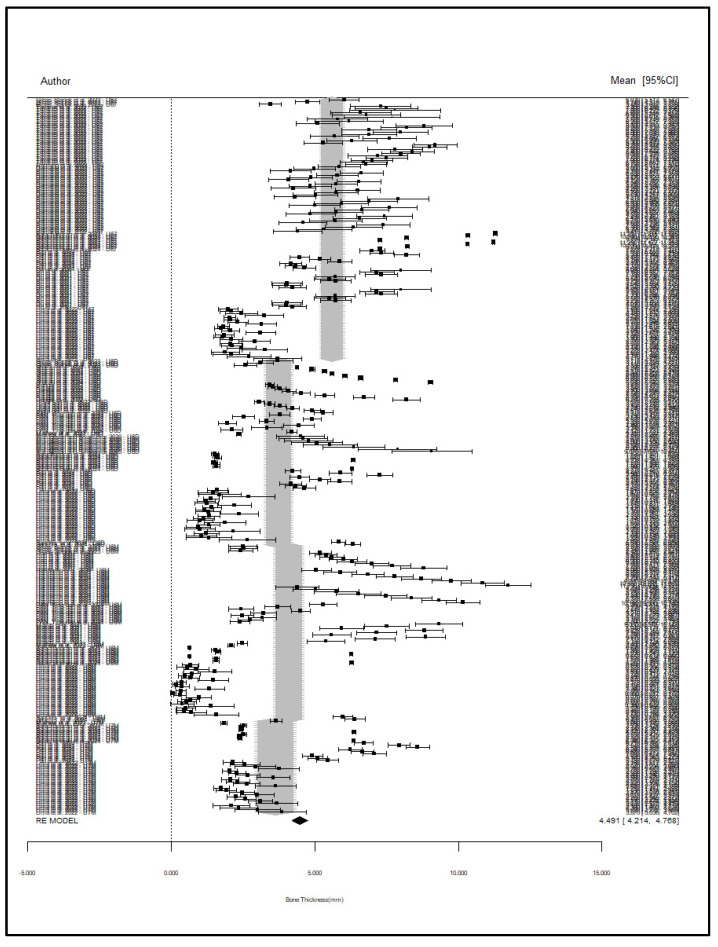
Overall Forest plot [1,5,10,11,25,26,27,28,29,30,31,32,33,34,35,36,37].

Provided that there are 331 subgroups to be plotted, this graph (Figure 6) is poorly intuitive. However, it is interesting to see the relative location of the estimation of the bone thickness for each position (grey surfaces).

**Figure 7:**
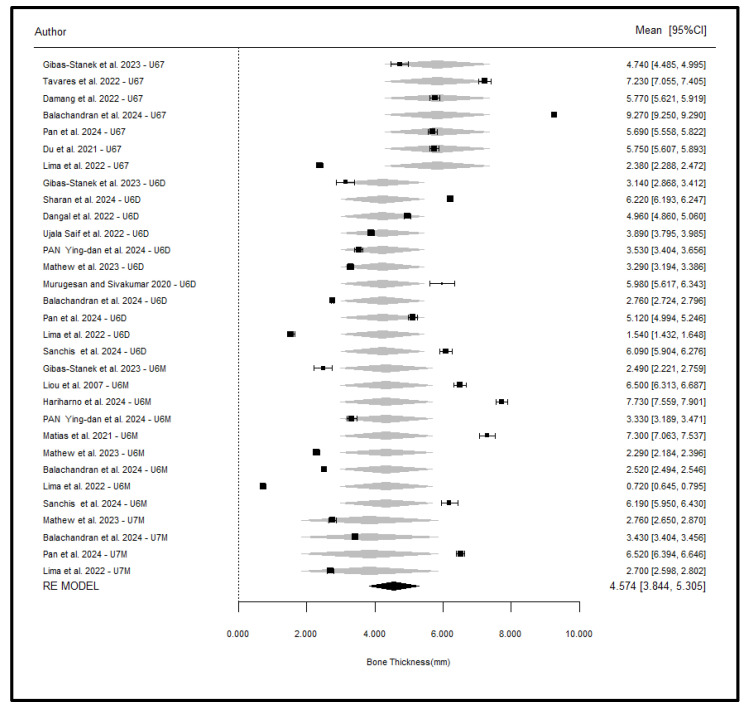
Average of all the subgroups of the same position [1,5,10,11,25,26,27,28,29,30,31,32,33,34,35,36,37].

When averaging all the subgroups for the same position within each article, a better approach was found, in which only the differences in position are shown (Figure 7).

It must be taken into account that this last meta-analysis is an approximation to the one estimated in (Figure 6), but the overall measure effect (4.57) is now only 1.7% higher than the previous one. It can be noticed how group U67 is present as a differentiated group from the other three groups (these are very similar). Additionally, confidence intervals are now narrower because the number of images/miniscrews has been added.

### 5.5. Multivariate Model for Height, Angle, and Position

A multivariate meta-regression analysis was used to assess the combined effects of height, angulation, and miniscrew position on bone thickness. The overall model explained 27.8% of the variance in bone thickness (R^2^ = 27.8%) and yielded results consistent with previous univariate models. The height was a strong and significant predictor (β = −0.42, *p* < 0.001), which means that for every 1 mm increase in insertion height, the bone thickness decreased by 0.42 mm, controlling for angulation and position. Angulation also showed a significant positive association with bone thickness (β = 0.05, *p* = 0.001), meaning each 1º increase in insertion angle was associated with a 0.05 mm increase in thickness. Concerning position, only the U67 location showed a statistically significant increase in bone thickness compared to the reference U6M (β = 1.37, *p* < 0.001). No significant differences were found for U6D (*p* = 0.921) or U7M (*p* = 0.876). Comparisons between U67 and other positions further confirmed these results, with significantly greater bone thickness in U67 relative to both U6D and U7M (*p* < 0.001 for both). These findings emphasize the multifactorial nature of bone thickness and highlight the unique advantage of the U67 site for miniscrew placement (Table 9).

As expected, R2 increased compared to simple models, but a relevant amount of between-studies heterogeneity remained (it should be explained by other variables not considered in the model).

The equation of regression (based in the first, with U6M as reference) expresses as follows:

BT=6.94−0.42 Height+0.05 Angle+0.03 U6D+1.37 U67−0.07 U7M

where U6D = 0 or 1, U67 = 0 or 1, and U7M = 0 or 1, depending on the position of the measurement.

The equation shows that the maximum bone thickness occurs when the miniscrew is placed in the U67 position with the observed height value of 9.9 mm and the angulation of 80º. Under these conditions, the equation predicts the highest bone thickness, which supports the clinical relevance of U67 as a favorable site for achieving optimal bone support in orthodontic miniscrew placement.

### 5.6. Effect of Facial Pattern

A basic meta-regression model analyzed the relationship between facial patterns (mesofacial, brachyfacial, dolicofacial) and bone thickness. The analysis showed no statistically relevant impact of facial pattern on bone thickness (overall *p* = 0.878) with an R^2^ of 0.0%, indicating that facial type failed to explain any study variance. The analysis revealed no important differences between brachyfacial (β = 0.16, *p* = 0.760) and dolicofacial (β = 0.27, *p* = 0.611) patterns when compared to the reference mesofacial group. The analysis revealed no meaningful differences between brachyfacial and dolicofacial individuals when the reference group was set to brachyfacial (*p* = 0.837). The current data indicates that facial growth patterns do not serve as significant predictors for infrazygomatic crest bone thickness (Table 10).

### 5.7. Multivariate Model for Height, Angle, Position, and Facial Pattern

**Figure 8:**
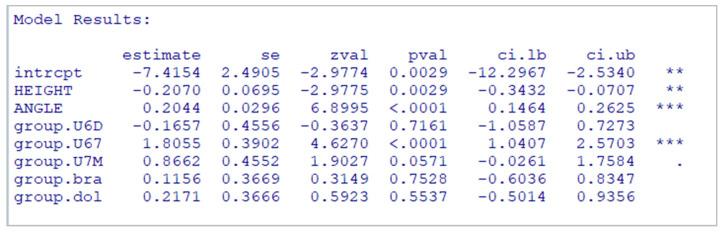
Model results for height, angle, position, and facial pattern. ** *p* < 0.01; *** *p* < 0.001.

A final multivariate meta-regression was performed, including all available predictors—height, angulation, miniscrew position, and facial pattern—to better understand how these factors combined to affect bone thickness. This model, which was estimated only on studies that reported facial pattern data, was very similar to the earlier full model (Table 9), with some expected differences. Height was still a significant negative predictor (β = −0.27, *p* = 0.003), but the effect was slightly reduced. Angulation remained strongly positively correlated with bone thickness (β = 0.20, *p* < 0.001), and the U67 position again had a significantly increased bone thickness (β = 1.81, *p* < 0.001), with an even stronger effect than in the previous model. The U7M position was approaching statistical significance (β = 0.86, *p* = 0.057), suggesting a potentially meaningful trend. As with the simpler model results, facial pattern was not significant (*p* > 0.05 for all groups), indicating that it does not meaningfully contribute to explaining bone thickness variability. These findings support the dominant role of anatomical variables, such as height, angulation, and insertion position, over the skeletal facial type in predicting infrazygomatic crest bone thickness (Figure 8).

## 6. Discussion

This systematic review and meta-analysis was carried out to evaluate the bone thickness at different IZC insertion heights, positions, and angulations for IZ miniscrew placement. It combined a meta-analysis with descriptive data to determine the best characteristics. Multiple research studies have established the best insertion height and angulation methods to achieve maximum bone contact while minimizing clinical complications. Liou et al. [11] found that placing the screw at 16 mm with angles above 55° produced optimal results but warned that angles exceeding 75° might lead to root damage. Sharan et al. [10] observed bone thicknesses of between 6 and 9 mm at vertical positions of 14.5–16 mm and angle ranges from 55° to 75°. Du et al. [29] recommended inserting the screw at 13–15 mm with a 60–70° gingival angulation and 30° distal angulation while advising against 17 mm or 50° due to lower bone thickness.

Comparable findings were observed in other studies. The vertical level of Class III patients should be between 5 and 6 mm according to Damang et al. [25], with an angulation of between 55 and 70°, but 5 mm represents the most suitable height for Class I patients. Pan et al. [26] and Hariharno et al. [33] supported 13 mm and 12 mm, respectively, as optimal screw lengths for the U67 region when the angulation reaches 70°. The authors Murugesan and Sivakumar [30] recommended 12–17 mm and 65–70° insertion for Dravidian patients because these settings minimize mucosal trauma while strengthening screw stability. According to Dangal et al. [31], the preferred insertion of 13 mm at 70° deviated from Taiwanese and Indian standards because of different ethnic facial structures.

Other authors expanded this framework by introducing supplementary factors. Sanchis et al. [5] advised against using screws exceeding 12 mm length because this increased the chance of sinus penetration, thus they recommended ≤11 mm as a safe length. Gibas-Stanek et al. [34] measured bone thickness at 12 mm as 6.03 ± 2.64 mm but observed a significant decrease to 2.42 ± 2.16 mm at 16 mm, which underscores the need for strategic planning during deep insertion procedures. Balachandran et al. [32] recommended placing screws at 11 mm distance from the cemento-enamel junction between the first and second molars with a 70° angle as the safest approach but warned against positioning them past the second molar because of the lower bone density. Pan Ying-dan et al. [27] recorded the highest bone thickness at a 13 mm distance from the left U6D occlusal plane.

Bone morphology in the IZC region differs between ethnic populations. According to Matias et al. [1], Brazilian Afro-Caucasians show increased maxillary protrusion along with more prominent soft tissue, which could influence optimal placement locations. Ujala Saif et al. [28] showed that bone thickness in the IZ region differed between Pakistani ethnic groups, which led to recommendations for screw size adjustments. The combination of ethnic differences and age-related changes with anatomical variations requires CBCT-based individualized planning to achieve optimal results.

**Figure 9:**
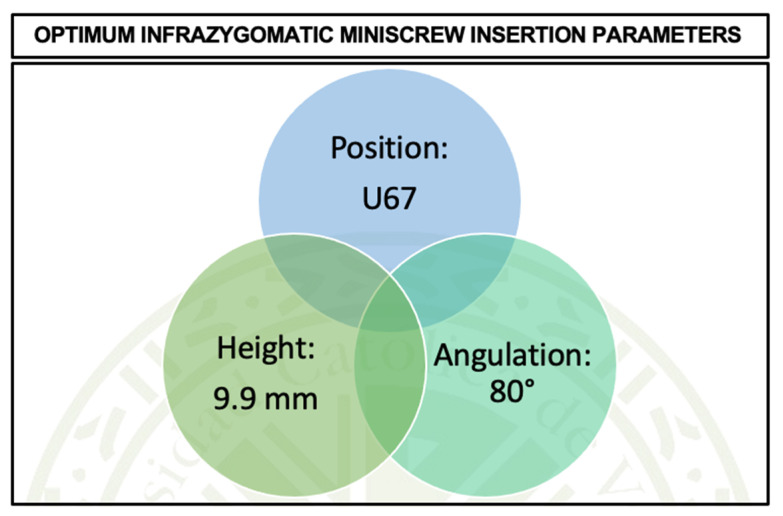
Optimum IZ miniscrew insertion parameters.

During the present study, the meta-regression analysis found statistical relationships between height, angulation, and position in relation to bone thickness, which can be useful in understanding the nature of the bone. Height was negatively correlated with bone thickness (*p* < 0.001), and for every 1 mm increase in height, the thickness reduced by 0.53 mm. Likewise, the angulation was positively correlated with bone thickness (*p* < 0.001), such that for every 1° increase in angulation, the bone thickness was increased by +0.09 mm. Anatomical factors were also found to be important in the positioning of miniscrews, and the U67 position had a greater bone thickness than the other positions (*p* < 0.001), particularly, U6M, U6D, and U7M. These results show that the height and position of the anatomy are important and should be taken into consideration when determining the bone thickness for clinical practice. However, there were moderate to high levels of heterogeneity in the analyses (I^2^ = 99.9%), which suggests that there are other unexamined factors that may account for the variation in bone thickness among individuals and studies. A multivariate meta-regression model that incorporated height, angulation, and position as explanatory variables explained 27.8% of the heterogeneity. Despite this enhancement, there was still a significant amount of residual heterogeneity (72.2%), which suggests that there are other unmeasured moderators, such as age, bone density, or material properties. The thickest bone was found at the U67 positions with the lowest heights and the highest angulation values (9.9 mm height, 80° angulation) (Figure 9). These results stress the importance of making individual evaluations during clinical practice, since both intrinsic (height, bone structure) and extrinsic (position, angulation) factors affect bone thickness. More anatomical and biomechanical factors should be investigated in future studies in order to enhance the prediction models and possibly explain the unexplained heterogeneity and enhance clinical practice in regard to miniscrew placement and bone thickness assessment.

The optimal placement of IZC miniscrews depends on treatment mechanics and individual anatomical differences, especially facial morphology [35]. Sanchis et al. reported that normodivergent patterns displayed shorter distances from the root apex to the sinus floor than hyperdivergent and hypodivergent groups, especially at the distobuccal root of second molars [5]. These findings are in agreement with Husseini et al., who observed that hyperdivergent patterns had the most variation in the height and depth at the mesiobuccal root of the maxillary first molars. This may be because of the decreased vertical dimension and the width of the maxillary sinus in hyperdivergent patterns, as well as a relatively larger maxillary alveolar ridge [39]. However, these results are inconsistent with Costea et al., who found that hypodivergent patterns had a shorter distance from the root apices to the maxillary sinus floor than hyperdivergent patterns [40].

The research by Lima et al. revealed safe zones (≥3 mm thickness) for hyperdivergent patients at 9–11 mm from the alveolar crest between the first and second molars, with the highest averages of 3.69 mm (right) and 3.87 mm (left). Neutral and hypodivergent groups displayed analogous patterns, with their highest averages at 3.64 mm (neutral) and 3.76 mm/3.56 mm (hypodivergent) at the mesial root. The bone thickness grew thicker as the measurement moved distally and apically, which indicated safer insertion points in the IZC region more apically [35]. Matias et al. predicted that brachyfacial patients would have larger IZC bone dimensions than dolichofacial patients, yet their research revealed no meaningful differences between facial types [1]. Mathew et al. discovered that brachyfacial and dolichofacial types displayed equivalent IZC thicknesses above the distal root of the first molar (*p* = 0.001), with usable heights ranging from 13–15 mm at a 70° angle [36].

The authors Tavares et al. suggested that the screw depth should not surpass 7–8 mm for safety purposes and observed that Class II and mesofacial patterns demonstrated increased bone loss at steeper insertion angles. Their research suggested that additional studies should evaluate how stature, the BMI, hormones, and ethnicity affect IZC suitability [37].

A meta-regression analysis was conducted to examine the effect of facial patterns on bone thickness, and no significant influence of this variable was found. The results showed that there was no significant difference in bone thickness among the brachyfacial, mesofacial, and dolicofacial groups (*p* = 0.878), and the comparisons between the brachyfacial and mesofacial (*p* = 0.760) and the dolicofacial and mesofacial (*p* = 0.611) groups were also non-significant. Furthermore, when the reference category was changed, the results were still consistent, and no significant differences were found between the dolicofacial and brachyfacial patterns (*p* = 0.837). These results show that facial pattern does not act as a key determinant of bone thickness in the IZC region, according to the results of the included studies.

Subsequently, a multivariate meta-regression model including height, angulation, position, and facial pattern was carried out to examine the combined impact of these variables. Although the facial pattern was still non-significant, the estimates of the other variables differed slightly from those of the previous models. For instance, the effect of height was reduced to a less-negative beta, while that of angulation became more positive. Likewise, the effect of U67 on bone thickness was also enhanced, and that of U7M was also close to being significant (*p* = 0.057). However, the basic patterns of relationships seen in the earlier models were still mostly evident, implying that height, angulation, and position are still the main drivers of bone thickness. This suggests the anatomical and biomechanical factors while rejecting the facial pattern as a significant contributor to the variation in IZ bone thickness. Future work may concentrate on other factors or relationships between the current factors to develop better models and keep on reducing the unexplained heterogeneity.

## 7. Conclusions

The systematic review and meta-analysis revealed important associations between insertion variables and bone thickness in the infrazygomatic crest (IZC) area, which will be useful for miniscrew placement. A clear inverse relationship was found between the vertical insertion height and bone thickness, with each 1 mm increase in height resulting in a 0.42 mm decrease in bone thickness when angulation and position were kept constant. On the other hand, angulation had a positive correlation with bone thickness, where for every additional degree of the insertion angle, bone thickness increased by approximately 0.05 mm. Among the anatomical positions assessed, the U67 region had the best bone support, with a significantly higher cortical thickness than U6M (+1.37 mm), U6D (+1.33 mm), and U7M (+1.43 mm). From the meta-regression modeling, the best parameters to use to obtain the maximum bone thickness were found to be at the position U67, the insertion height of 9.9 mm, and the angulation of 80°. There was no significant association found between facial growth patterns and the IZC bone thickness, which means that skeletal divergence cannot be used as a reliable predictor for miniscrew insertion planning in this area. These findings provide significant evidence for the improvement of orthodontic anchorage strategies and highlight the need for further clinical research to validate these recommendations in patient-specific scenarios. However, there are some limitations of this study that should be taken into account. Most of the included studies were based on cross-sectional CBCT analyses, which do not consider dynamic clinical factors, such as patient-specific healing responses, soft tissue variability, or long-term screw stability. Furthermore, variations in the sample ethnicity and age, as well as in the imaging protocols used in the different studies, may have led to heterogeneity. The anatomical benchmarks from this meta-analysis help IZC miniscrew placement, but individual patient assessment remains essential. The use of CBCT-based planning for each patient’s unique anatomy leads to safe and accurate insertion, which supports personalized dental care. Further prospective clinical trials and longitudinal CBCT-based studies that incorporate patient-specific variables with clinical outcomes and standardized measurement protocols are required to establish the generalizability and practicality of these findings.

## Figures and Tables

**Table 1:** Inclusion criteria.

Category	Inclusion Criteria
Type of Study	Quantitative, cross-sectional, observational studies and retrospective studies utilizing Cone Beam Computed Tomography (CBCT) imaging, published in any language between January 2000 and January 2025.
Type of Patients	Patients had to have all permanent teeth visible radiographically (excluding third molars) and healthy periodontal conditions and no maxillary sinus problems when imaging occurred.
Type of Intervention	Bone thickness in different infrazygomatic crest (IZC) regions at various insertion heights and angles and mesio-distal positions.
Type of Outcome Variables	Studies reporting quantitative data about IZC bone thickness that could be statistically analyzed through means and standard deviations and insertion parameters.

**Table 2:** Exclusion criteria.

Category	Exclusion Criteria
Type of Study	Case reports, in vitro or finite element studies, literature reviews, cephalometric-only analyses, and studies lacking CBCT-based anatomical measurements.
Type of Patient	Individuals with mixed or deciduous dentition and those who had existing implants or prostheses in the posterior maxilla (that may lead to artifacts in imaging), medications that can affect bone health, or systemic conditions that affect bone metabolism.
Type of Intervention	Interventions that did not address IZC specific screw insertion or those that focused on other anchorage sites, such as the mandibular buccal shelf or zygomatic plates.
Type of Outcome Variables	Articles lacking detailed measurements of bone thickness or presenting insufficient statistical data.

**Table 3:** Summary of keywords and Boolean operators used in each database.

DATABASE	KEYWORDS
EBSCO	“Infrazygomatic” OR “zygomatic crest” AND “miniscrew” AND “design or size” OR “insertion angle” AND “CBCT”
PUBMED	(((((infrazygomatic) OR (zygomatic crest)) AND (miniscrew)) AND (design or size)) OR (insertion angle)) AND (cbct)
SCOPUS	“Infrazygomatic” OR “zygomatic” AND “crest” AND “miniscrew” AND “design” OR “size” OR “insertion” AND “angle” AND “CBCT”
COCHRANE	“Infrazygomatic” OR “Zygomatic crest” AND “miniscrew” AND “CBCT”
GOOGLE SCHOLAR	“infrazygomatic”,“CBCT”, “Miniscrew”, “size”, “design”, “insertion”
WEB OF SCIENCE	“Infrazygomatic” OR “zygomatic crest” AND “miniscrew” AND “design or size” OR “insertion angle” AND “CBCT”

**Table 4:** Information and data extracted from articles for meta-analysis.

Article	Author	Year	Insertion Height (± Standard Deviation (If Present)	Gingival Inclinication	Insertion Region	Mean Bone Thickness at 0° Mesio-Distal Inclination	Standard Deviation for Mean Bone Thickness	Median—With 0° Mesio-Distal Inclination (25th to 75th Percentile)	*p*	Quartiles	Mean Bone Thickness at a Specified Mesio-Distal (If Used) ± Standard Deviation	
											15°	30°
Quantitative Evaluation of the Infrazygomatic Crest Thickness in Polish Subjects: A Cone-Beam Computed Tomography Study	Gibas-Stanek et al.	2023	12 mm	70°	U6M	2.5	2.55	2.13	*p* < 0.001 U67 > U6D > U6M	0–3.9		
					U6D	3.71	2.76	3.53		1.53–5.23		
					U67	6.03	2.64	5.94		4.25–7.65		
			14 mm	70°	U6M	2.54	2.42	2.36	*p* < 0.001 U67 > U6D > U6M	0–4.02		
					U6D	3.11	2.35	3.29		0.86–4.64		
					U67	4.74	2.17	4.84		3.04–6.17		
			16 mm	70°	U6M	2.42	2.16	2.16	*p* = 0.453	0–3.95		
					U6D	2.59	2.08	2.29		1–3.65		
					U67	3.46	1.93	3.54		1.83–4.7		
Assessment of Bone Thickness at the Infra Zygomatic Crest Region for Various Orthodontic Miniscrew Implant (OMSI) Insertion Angles: A Cone-Beam Computed Tomographic Study	Sharan et al.	2024	17.71 ± 0.61 mm	40°	U6D	4.39	0.25		0.531			
			17.04 ± 0.53 mm	45°		4.91	0.45		0.982			
			16.28 ± 0.56 mm	50°		5.34	0.40		0.763			
			16.10 ± 0.52 mm	55°		5.60	0.40		0.960			
			15.54 ± 0.71 mm	60°		6.06	0.41		0.806			
			15.03 ± 0.71 mm	65°		6.60	0.41		0.159			
			14.37 ± 0.62 mm	70°		7.82	0.33		0.880			
			13.69 ± 0.75 mm	75°		9.03	0.45		0.577			
Comparison of Bone Thickness in Infrazygomatic Crest Area at Various Miniscrew Insertion Angles: A Cone-beam Computed Tomographic Study	Dangal et al.	2022	17.07 ± 1.90 mm	40°	U6D	3.44	0.75					
			16.71 ± 1.94 mm	45°		3.54	0.80					
			16.27 ± 1.99 mm	50°		3.80	0.93					
			16.05 ± 3.47 mm	55°		4.10	1.17					
			15.16 ± 2.72 mm	60°		4.54	1.42					
			14.28 ± 2.72 mm	65°		5.35	1.66					
			12.99 ± 2.89 mm	70°		6.72	1.80					
			11.26 ± 2.65 mm	75°		8.20	2.26					
A computed tomographic image study on the thickness of the infrazygomatic crest of the maxilla and its clinical implications for miniscrew insertion	Liou et al.	2007	17.1 ± 3.7 mm	40°	U6M	5.2	1.1		* *p* < 0.001			
			16.8 ± 3.7 mm	45°		5.4	1.1					
			16.6 ± 3.4 mm	50°		5.6	1.2					
			16.1 ± 3.5 mm	55°		6.0	1.4					
			15.4 ± 3.5 mm	60°		6.3	1.5					
			14.7 ± 3.6 mm	65°		7.0	1.7					
			13.8 ± 3.8 mm	70°		7.7	1.9					
			12.8 ± 4.2 mm	75°		8.8	2.3					
Optimal Placement Site and Angulation for Infrazygomatic Screw’s Insertion—A CBCT Study	Hariharno et al.	2024	14.26 ± 1.47 mm	40°	U6M	5.06	1.44		0.001			
			13.79 ± 1.54 mm	45°		5.89	1.62					
			13.37 ± 1.38 mm	50°		6.86	1.59					
			12.89 ± 1.44 mm	55°		7.78	1.78					
			12.24 ± 1.39 mm	60°		8.72	1.94					
			11.56 ± 1.66 mm	65°		9.76	1.89					
			10.87 ± 1.86 mm	70°		10.85	2.11					
			10.17 ± 1.82 mm	75°		11.73	2.21					
			14.08 ± 1.50 mm	40°	U6M	4.39	2.08		0.05			
			13.58 ± 1.6 mm	45°		5.04	2.19					
			13.01 ± 1.4 mm	50°		5.76	2.18					
			12.41 ± 1.77 mm	55°		6.53	2.19					
			11.82 ± 1.83 mm	60°		7.48	2.06					
			11.07 ± 1.88 mm	65°		8.37	1.99					
			10.38 ± 2.19 mm	70°		9.33	1.7					
			9.9 ± 2.07 mm	75°		10.16	1.62					
Assesment of infrazygomatic bone thickness for safe placement of infrazygomatic implant in pakistani population; a CBCT study.	Ujala Saif et al.	2022	18.40 ± 2.99	55°	U6D	3.07	0.94		0.74			
			17.86 ± 3.05	60°		3.43	1.00		0.61			
			17.37 ± 3.13	65°		3.79	1.10		0.88			
			16.79 ± 3.29	70°		4.24	1.24		0.62			
			16.02 ± 3.59	75°		4.91	1.56		0.58			
An evaluation of infrazygomatic crest bone thickness in adolescents at different eruption stages of the maxillary second molar detected by cone beam CT	PAN Ying-dan et al.	2024	13 mm	60°	U6M	5.29	1.61	5.01 (4.30–6.27)	<0.001			
			15 mm			3.70	1.53	3.44 (2.55–4.58)	0.007			
			17 mm			2.44	1.51	1.96 (1.32–3.50)	0.779			
			13 mm	60°	U6D	5.31	1.12	5.00 (4.24–5.48)	0.001			
			15 mm			3.79	1.19	3.35 (2.71–3.98)	0.110			
			17 mm			2.53	1.31	1.90 (1.28–2.70)	0.140			
			13 mm	60°	U6M	4.51	0.91	4.68 (3.63–5.32)	<0.001			
			15 mm			3.21	1.26	3.23 (2.20–4.16)	0.007			
			17 mm			2.47	1.11	2.46 (1.55–3.26)	0.779			
			13 mm	60°	U6D	4.92	0.87	5.00 (4.24–5.48)	0.001			
			15 mm			3.33	0.71	3.35 (2.71–3.98)	0.110			
			17 mm			1.96	0.87	1.90 (1.28–2.70)	0.140			
			13 mm	60°	U6M	3.18	1.48	3.15(1.73–4.07)	<0.001			
			15 mm			2.76	1.34	2.55(1.77–3.38)	0.007			
			17 mm			2.39	1.05	2.12(1.44–3.32)	0.779			
			13 mm	60°	U6D	4.46	1.63	3.96 (3.54–5.40)	0.001			
			15 mm			3.34	1.64	3.25 (2.11–3.96)	0.110			
			17 mm			2.12	1.19	1.92 (1.14–2.66)	0.140			
Miniscrew insertion sites of infrazygomatic crest and mandibular buccal shelf in different vertical craniofacial patterns: A cone-beam computed tomography study	Matias et al.	2021	11 mm	70°	U6M	9.33	2.27		0.565			
			13 mm			7.51	2.16		0.712			
			15 mm			5.94	2.15		0.561			
			11 mm	70°	U6M	8.82	1.83		0.565			
			13 mm			7.16	1.93		0.712			
			15 mm			5.59	1.88		0.561			
			11 mm	70°	U6M	8.87	1.91		0.565			
			13 mm			7.11	1.95		0.712			
			15 mm			5.39	1.86		0.561			
Three-dimensional Comparison of Infra-zygomatic Crest Thickness in Different Facial Patterns: A Cross-sectional Study	Mathew et al.	2023	13.67 ± 2.09	70°	U6M	2.48	0.67		0.001			
			15.27 ± 1.10		U6D	4.21	0.63					
			14.45 ± 1.08		U7M	3.66	0.72					
			15.48 ± 1.04	70°	U6M	2.09	0.38		0.001			
			15.35 ± 0.97		U6D	2.36	0.33					
			15.71 ± 1.07		U7M	1.85	0.38					
Comparison of bone thickness in infrazygomatic crest area at various miniscrew insertion angles in Dravidian population—A cone beam computed tomography study	Murugesan and Sivakumar	2020	17.31 ± 1.68 mm	40°	U6D	4.51	1.92		0.001			
			16.92 ± 1.69 mm	45°		4.60	1.95					
			16.58 ± 1.65 mm	50°		4.80	1.94					
			16.07 ± 1.63 mm	55°		5.08	2.02					
			15.46 ± 1.71 mm	60°		5.54	2.14					
			14.46 ± 2.08 mm	65°		6.36	2.47					
			12.83 ± 2.70 mm	70°		7.89	3.12					
			11.43 ± 2.74 mm	75°		9.07	3.18					
Tomographic assessment of infrazygomatic crest bone depth for extra-alveolar miniscrew insertion in subjects with different vertical and sagittal skeletal patterns	Tavares et al.	2022	4 + (7.5) mm	60°	U67	8.7	3.1		(a) 60° at 4 mm and 70° at 6 mm and 80° at 5 and 6 mm (*p* = 0.004, *p* = 0.005 and *p* = 0.000, respectively). (b) 60° at 5 mm and 80° at 6 mm (*p* = 0.007). (c) 70° at 4 mm and 70° at 6 mm and 80° at 5 and 6 mm (*p* = 0.034; *p* = 0.041 and *p* = 0.000, respectively).			
				70°		8.4	2.8					
				80°		7.4	3.4					
			5 + (7.5) mm	60°	U67	7.9	3.1					
				70°		7.3	3.0					
				80°		6.6	3.4					
			6 + (7.5) mm	60°	U67	7.3	3.0					
				70°		6.5	2.9					
				80°		5.8	2.7					
			4 + (7.5) mm	60°	U67	7.3	2.4					
				70°		7.5	2.5					
				80°		7.8	3.6					
			5 + (7.5) mm	60°	U67	6.6	2.4					
				70°		6.8	2.7					
				80°		7.7	3.8					
			6 + (7.5) mm	60°	U67	5.8	2.4					
				70°		6.2	2.7					
				80°		5.1	1.7					
			4 + (7.5) mm	60°	U67	8.8	3.7		Significant difference (*p* < 0.05) between 60° at 4 mm and 80° at 5 and 6 mm.			
				70°		8.2	3.3					
				80°		6.9	3.6					
			5 + (7.5) mm	60°	U67	8.0	(3.6					
				70°		6.9	3.6					
				80°		5.7	3.2					
			6 + (7.5) mm	60°	U67	7.6	3.5					
				70°		6.3	3.3					
				80°		5.3	2.5					
			4 + (7.5) mm	60°	U67	9.2	2.5					
				70°		9.0	2.0					
				80°		7.8	3.2					
			5 + (7.5) mm	60°	U67	8.4	2.5					
				70°		8.0	2.2					
				80°		7.2	3.4					
			6 + (7.5) mm	60°	U67	7.5	2.4					
				70°		7.0	2.4					
				80°		6.7	2.7					
A Computed Tomographic Image Study on Thickness of the Modified Infrazygomatic Crest Site Between Patients with Class I and Class III Skeletal Pattern	Damang et al.	2022	5 + (7.5) mm	55°	U67	6.79	2.01		0.940			
			6 + (7.5) mm			5.86	2.07		0.127			
			7 + (7.5) mm			4.99	2.04		0.161			
			8 + (7.5) mm			4.16	2.01		0.161			
			5 + (7.5) mm	60°	U67	6.63	2.11		0.105			
			6 + (7.5) mm			5.79	2.06		0.170			
			7 + (7.5) mm			4.87	2.07		0.178			
			8 + (7.5) mm			4.10	1.97		0.333			
			5 + (7.5) mm	65°	U67	6.54	2.16		0.126			
			6 + (7.5) mm			5.75	2.04		0.146			
			7 + (7.5) mm			4.85	2.08		0.153			
			8 + (7.5) mm			4.26	2.15		0.644			
			5 + (7.5) mm	70°	U67	6.50	2.17		0.156			
			6 + (7.5) mm			5.74	2.14		0.195			
			7 + (7.5) mm			5.03	2.16		0.555			
			8 + (7.5) mm			4.31	2.03		0.871			
			5 + (7.5) mm	55°	U67	7.91	2.99		0.940			
			6 + (7.5) mm			6.9	2.77		0.127			
			7 + (7.5) mm			5.93	2.64		0.161			
			8 + (7.5) mm			5.00	2.49		0.161			
			5 + (7.5) mm	60°	U67	7.60	2.73		0.105			
			6 + (7.5) mm			6.64	2.64		0.170			
			7 + (7.5) mm			5.76	2.58		0.178			
			8 + (7.5) mm			4.85	2.40		0.333			
			5 + (7.5) mm	65°	U67	7.43	2.69		0.126			
			6 + (7.5) mm			6.57	2.57		0.146			
			7 + (7.5) mm			5.73	2.57		0.153			
			8 + (7.5) mm			4.60	2.33		0.644			
			5 + (7.5) mm	70°	U67	7.39	2.63		0.156			
			6 + (7.5) mm			6.36	2.77		0.195			
			7 + (7.5) mm			5.32	2.7		0.555			
			8 + (7.5) mm			4.41	2.38		0.871			
Evaluation Of Bone Thickness At Different Anatomical Sites In Infrazygomatic Crest For Miniscrew Insertion In Skeletal Class II Patients—A CBCT Study	Balachandran et al.	2024	5 + (7.5) mm	70°	U6M	0.64	0.03		0.000			
			7 + (7.5) mm			1.56	0.37		0.000			
			9 + (7.5) mm			1.63	0.22		0.000			
			11 + (7.5) mm			6.27	0.14		0.000			
			5 + (7.5) mm	70°	U6D	1.52	0.29		0.000			
			7 + (7.5) mm			1.57	0.29		0.000			
			9 + (7.5) mm			1.64	0.29		0.000			
			11 + (7.5) mm			6.33	0.16		0.000			
			5 + (7.5) mm	70°	U67	11.28	0.16		0.000			
			7 + (7.5) mm			10.34	0.13		0.000			
			9 + (7.5) mm			8.20	0.15		0.000			
			11 + (7.5) mm			7.27	0.13		0.000			
			5 + (7.5) mm	70°	U7M	2.53	0.26		0.000			
			7 + (7.5) mm			2.44	0.18		0.000			
			9 + (7.5) mm			2.42	0.17		0.000			
			11 + (7.5) mm			6.37	0.13		0.000			
			5 + (7.5) mm	70°	U6M	0.65	0.04		0.000			
			7 + (7.5) mm			1.57	0.25		0.000			
			9 + (7.5) mm			1.57	0.25		0.000			
			11 + (7.5) mm			6.28	0.17		0.000			
			5 + (7.5) mm	70°	U6D	1.51	0.27		0.000			
			7 + (7.5) mm			1.58	0.28		0.000			
			9 + (7.5) mm			1.58	0.27		0.000			
			11 + (7.5) mm			6.31	0.13		0.000			
			5 + (7.5) mm	70°	U67	11.23	0.13		0.000			
			7 + (7.5) mm			10.33	0.11		0.000			
			9 + (7.5) mm			8.23	0.14		0.000			
			11 + (7.5) mm			7.27	0.14		0.000			
			5 + (7.5) mm	70°	U7M	2.53	0.25		0.000			
			7 + (7.5) mm			2.37	0.13		0.000			
			9 + (7.5) mm			2.39	0.16		0.000			
			11 + (7.5) mm			6.36	0.13		0.000			
An evaluation of bone depth at different three-dimensional paths in infrazygomatic crest region for miniscrew insertion: A cone beam computed tomography study	Pan et al.	2024	13 mm	60°	U6D	4.24	1.79	3.96 (3.15–5.20)			5.27 ± 2.47	7.07 ± 3.56
				70°		5.90	2.48	5.71 (4.26–7.31)			6.78 ± 3.04	8.49 ± 3.62
				80°		7.26	2.87	7.08 (5.42–8.58)			7.97 ± 3.16	9.64 ± 3.80
			15 mm	60°		4.48	2.25	4.09 (3.11–5.25)			4.90 ± 2.67	5.99 ± 3.31
				70°		5.19	2.44	4.63 (3.65–6.16)			5.58 ± 2.85	6.57 ± 3.36
				80°		5.88	2.73	5.38 (4.31–7.01)			6.16 ± 2.92	7.41 ± 3.57
			17 mm	60°		4.17	2.46	3.44 (2.74–4.68)			mean = 4.17 ± 2.59. -MINIMUM BONE DEPTH (median (25th to 75th) = 3.40 (2.62–4.83))	4.59 ± 2.87
				70°		4.34	2.36	3.77 (2.80–5.13)			4.36 ± 2.54	4.98 ± 3.11
				80°		4.64	2.48	4.07 (2.96–5.49)			4.69 ± 2.67	5.43 ± 3.28
			13 mm	60°	U67	7.00	2.87	6.44 (5.25–8.19)			7.63 ± 3.12	7.67 ± 2.69
				70°		7.33	2.63	6.78 (5.58–8.49)			8.20 ± 2.79	9.50 ± 3.56
				80°		8.20	2.79	7.29 (6.05–9.06)			8.89 ± 3.11	10.80 ± 3.89
			15 mm	60°		4.48	2.25	4.97 (4.10–6.73)			6.14 ± 2.98	6.80 ± 3.09
				70°		5.19	2.44	5.29 (4.32–7.00)			6.34 ± 2.78	7.62 ± 3.59
				80°		5.88	2.73	5.81 (4.60–7.48)			6.84 ± 2.96	8.45 ± 3.83
			17 mm	60°		4.17	2.46	3.90 (2.91–5.11)			4.51 ± 2.64	5.09 ± 3.03
				70°		4.34	2.36	4.01 (3.03–5.39)			4.69 ± 2.51	5.51 ± 3.32
				80°		4.64	2.48	4.31 (3.23–5.68)			5.06 ± 2.68	6.03 ± 3.38
			13 mm	60°	U7M	6.71	2.09	6.51 (5.34–7.75)			7.77 ± 2.71	8.08 ± 3.51
				70°		7.94	2.49	7.65 (6.24–9.37)			9.14 ± 2.76	10.18 ± 4.13
				80°		8.58	2.65	8.15 (6.80–10.15)			10.01 ± 3.20	Mean = 11.69 ± 3.95—MAXIMUM BONE DEPTH (median (25th to 75th) = 10.72 (8.89–14.05))
			15 mm	60°		6.24	2.60	5.63 (4.55–7.16)			6.80 ± 3.76	7.81 ± 3.62
				70°		6.66	2.50	6.23 (4.89–8.21)			7.37 ± 2.83	8.84 ± 3.79
				80°		7.08	2.62	6.72 (5.21–8.68)			8.04 ± 3.08	9.84 ± 3.94
			17 mm	60°		4.92	2.29	4.55 (3.36–6.17)			5.20 ± 2.50	6.32 ± 3.56
				70°		5.10	2.24	4.68 (3.53–6.42)			5.52 ± 2.67	6.90 ± 3.78
				80°		5.45	2.40	5.16 (3.74–6.78)			6.04 ± 2.87	7.58 ± 3.84
Bone depth and thickness of different infrazygomatic crest miniscrew insertion paths between the first and second maxillary molars for distal tooth movement: A 3-dimensional assessment	Du et al.	2021	13 mm	50°	U67	8.00	4.54	6.70 (5.16–8.47)			7.82 ± 3.85	8.42 ± 4.10
				60°		7.16	2.61	6.56 (5.29–8.24)			7.19 ± 2.56	7.45 ± 2.62
				70°		7.33	2.44	6.81 (5.56–8.34)			7.31 ± 2.39	7.40 ± 2.40
			15 mm	50°		5.72	2.92	5.01 (3.60–6.57)			5.76 ± 2.96	6.34 ± 3.64
				60°		5.52	2.38	5.02 (3.82–6.39)			5.54 ± 2.34	5.75 ± 2.41
				70°		5.73	2.28	5.23 (4.02–6.74)			5.72 ± 2.24	5.80 ± 2.27
			17 mm	50°		4.04	2.32	3.49 (2.41–4.94)			4.11 ± 2.31	4.38 ± 2.45
				60°		4.02	2.12	3.53 (2.53–4.77)			4.05 ± 2.07	4.20 ± 2.16
				70°		4.24	2.08	3.75 (2.67–5.07)			4.26 ± 2.09	4.32 ± 2.10
			13 mm	50°	U67	8.00	4.54	6.70 (5.16–8.47)			7.82 ± 3.85	8.42 ± 4.10
				60°		7.16	2.61	6.56 (5.29–8.24)			7.19 ± 2.56	7.45 ± 2.62
				70°		7.33	2.44	6.81 (5.56–8.34)			7.31 ± 2.39	7.40 ± 2.40
			15 mm	50°		5.72	2.92	5.01 (3.60–6.57)			5.76 ± 2.96	6.34 ± 3.64
				60°		5.52	2.38	5.02 (3.82–6.39)			5.54 ± 2.34	5.75 ± 2.41
				70°		5.73	2.28	5.23 (4.02–6.74)			5.72 ± 2.24	5.80 ± 2.27
			17 mm	50°		4.04	2.32	3.49 (2.41–4.94)			4.11 ± 2.31	4.38 ± 2.45
				60°		4.02	2.12	3.53 (2.53–4.77)			4.05 ± 2.07	4.20 ± 2.16
				70°		4.24	2.08	3.75 (2.67–5.07)			4.26 ± 2.09	4.32 ± 2.10
Safe sites for orthodontic miniscrew insertion in the infrazygomatic crest area in different facial types: A tomographic study	Lima et al.	2022	5 + (8.01) mm	(50–70)°	U6M	0.68	0.86		<0.001			
			7 + (8.01) mm			0.57	1.05					
			9 + (8.01) mm			0.88	1.29					
			11 + (8.01) mm			1.53	1.7					
			5 + (8.01) mm	(50–70)°	U6D	1.61	1.02		0.002			
			7 + (8.01) mm			1.47	1.57					
			9 + (8.01) mm			1.69	2.13					
			11 + (8.01) mm			2.7	2.65					
			5 + (8.01) mm	(50–70)°	U67	1.99	1		<0.001			
			7 + (8.01) mm			2.1	1.11					
			9 + (8.01) mm			2.45	1.61					
			11 + (8.01) mm			3.25	1.94					
			5 + (8.01) mm	(50–70)°	U7M	2.16	0.99		<0.001			
			7 + (8.01) mm			2.57	1.49					
			9 + (8.01) mm			2.94	2.05					
			11 + (8.01) mm			3.76	2.04					
			5 + (8.01) mm	(50–70)°	U6M	0.72	0.88		0.002			
			7 + (8.01) mm			0.49	0.71					
			9 + (8.01) mm			0.67	0.9					
			11 + (8.01) mm			1.47	1.55					
			5 + (8.01) mm	(50–70)°	U6D	1.4	0.76		0.001			
			7 + (8.01) mm			1.23	0.91					
			9 + (8.01) mm			1.24	1.18					
			11 + (8.01) mm			2.21	1.69					
			5 + (8.01) mm	(50–70)°	U67	2.04	0.57		<0.001			
			7 + (8.01) mm			2.07	0.68					
			9 + (8.01) mm			2.33	1.05					
			11 + (8.01) mm			3.15	1.54					
			5 + (8.01) mm	(50–70)°	U7M	2.05	0.75		<0.001			
			7 + (8.01) mm			2.3	0.88					
			9 + (8.01) mm			2.68	1.24					
			11 + (8.01) mm			3.56	1.68					
			5 + (8.01) mm	(50–70)°	U6M	0.37	0.45		<0.001			
			7 + (8.01) mm			0.19	0.36					
			9 + (8.01) mm			0.38	0.69					
			11 + (8.01) mm			1.34	1.45					
			5 + (8.01) mm	(50–70)°	U6D	1.42	0.91		0.001			
			7 + (8.01) mm			1.17	0.89					
			9 + (8.01) mm			1.3	1.21					
			11 + (8.01) mm			2.37	1.86					
			5 + (8.01) mm	(50–70)°	U67	1.83	0.59		<0.001			
			7 + (8.01) mm			1.74	0.67					
			9 + (8.01) mm			2.06	0.88					
			11 + (8.01) mm			3.12	1.45					
			5 + (8.01) mm	(50–70)°	U7M	2.07	0.84		<0.001			
			7 + (8.01)mm			2.32	1.15					
			9 + (8.01) mm			2.64	1.3					
			11 + (8.01) mm			3.64	2.01					
			5 + (8.01) mm	(50–70)°	U6M	0.35	0.52		<0.001			
			7 + (8.01) mm			0.08	0.27					
			9 + (8.01) mm			0.31	0.64					
			11 + (8.01) mm			0.97	1.21					
			5 + (8.01) mm	(50–70)°	U6D	1.33	1.06		0.020			
			7 + (8.01) mm			1.14	1.04					
			9 + (8.01) mm			1.01	1.28					
			11 + (8.01) mm			1.88	2.06					
			5 + (8.01) mm	(50–70)°	U67	1.86	0.9		<0.001			
			7 + (8.01) mm			1.8	0.85					
			9 + (8.01) mm			2.1	1.1					
			11 + (8.01) mm			2.91	1.53					
			5 + (8.01) mm	(50–70)°	U7M	1.75	0.77		<0.001			
			7 + (8.01) mm			1.93	0.98					
			9 + (8.01) mm			2.47	1.23					
			11 + (8.01) mm			3.01	1.63					
			5 + (8.01) mm	(50–70)°	U6M	0.68	0.78		0.008			
			7 + (8.01) mm			0.55	0.83					
			9 + (8.01) mm			0.39	1.03					
			11 + (8.01) mm			1.39	1.98					
			5 + (8.01) mm	(50–70)°	U6D	1.32	0.97		0.006			
			7 + (8.01) mm			0.94	1.06					
			9 + (8.01)mm			1	1.35					
			11 + (8.01)mm			2.18	2.32					
			5 + (8.01)mm	(50–70)°	U67	2.08	0.94		0.002			
			7 + (8.01) mm			2.13	0.91					
			9 + (8.01) mm			2.45	1.26					
			11 + (8.01) mm			3.27	1.99					
			5 + (8.01) mm	(50–70)°	U7M	2.26	0.78		<0.001			
			7 + (8.01) mm			2.59	0.91					
			9 + (8.01) mm			3.11	1.09					
			11 + (8.01) mm			3.69	1.8					
			5 + (8.01) mm	(50–70)°	U6M	0.52	0.81		0.001			
			7 + (8.01) mm			0.46	0.74					
			9 + (8.01) mm			0.71	1.29					
			11 + (8.01) mm			1.58	1.98					
			5 + (8.01) mm	(50–70)°	U6D	1.23	1.09		<0.001			
			7 + (8.01) mm			1.06	1.3					
			9 + (8.01) mm			1.32	1.95					
			11 + (8.01) mm			2.66	2.45					
			5 + (8.01) mm	(50–70)°	U67	1.89	1.25		<0.001			
			7 + (8.01) mm			2.1	1.58					
			9 + (8.01) mm			2.71	1.91					
			11 + (8.01) mm			3.71	2.1					
			5 + (8.01) mm	(50–70)°	U7M	2.1	1.58		<0.001			
			7 + (8.01) mm			2.36	1.67					
			9 + (8.01) mm			3.02	1.97					
			11 + (8.01) mm			3.87	2.08					
Optimal sites for mini-implant insertion into the infrazygomatic crest according to different craniofacial morphologies: A cross-sectional cone-beam computed tomography study	Sanchis et al.	2024		60°	U6M	5.98	2.38	5.66 (4.00–7.58)	*p* <0.001			
				60°	U6D	5.84	1.80	5.86 (4.50–7.01)	*p* <0.001			
				60°	U6M	6.40	2.53	6.00 (4.51–8.20)	*p* <0.001			
				60°	U6D	6.33	1.99	6.34 (4.80–7.68)	*p* <0.001			

**Table 5:** Keywords of information and data extracted from articles for the meta-analysis.

KEYWORDS
U6M: Mesiobuccal root of the first maxillary molar.
U6D: Distobuccal root of the first maxillary molar.
U67: Between the maxillary first and the maxillary second molar.
U7M: Mesiobuccal root of the maxillary second molar.

**Table 6:** Results of meta-regression of bone thickness by height: beta coefficient, standard error (SE), 95% confidence interval, z test (*p*-value), and R^2^.

	Beta	SE	95% CI	z	* p * -Value	R^2^
intercept	12.3	0.89	10.6 × 14.1	13.8	<0.001 ***	
HEIGHT	−0.53	0.06	−0.64 × −0.41	−8.91	<0.001 ***	19.2%

*** *p* < 0.001.

**Table 7:** Results of meta-regression of bone thickness by angulation: beta coefficient, standard error (SE), 95% confidence interval, z test (*p*-value), and R^2^.

	Beta	SE	95% CI	z	*p*-Value	R^2^
intercept	−1.28	1.10	−3.45 × 0.88	−1.16	0.246	
ANGULATION	0.09	0.02	0.06 × 0.13	5.27	<0.001 ***	7.54%

*** *p* < 0.001.

**Table 8:** Results of meta-regression of bone thickness by position: beta coefficient, standard error (SE), 95% confidence interval, z test (*p*-value) and R^2^.

	Beta	SE	95% CI	z	* p * -Value	R^2^
intercept	4.09	0.27	3.57 × 4.62	15.2	<0.001 ***	10.4%
U6D	−0.36	0.38	−1.09 × 0.38	−0.96	0.337	
U67	1.51	0.35	0.83 × 2.20	4.33	<0.001 ***	
U7M	−0.46	0.46	−1.35 × 0.44	−0.99	0.319	

*** *p* < 0.001.

**Table 9:** Results of meta-regression of bone thickness by height, angulation and position: beta coefficient, standard error (SE), 95% confidence interval, z test (*p*-value), and R^2^.

	Beta	SE	95% CI	z	* p * -Value	R^2^
intercept	6.94	1.51	3.98 × 9.89	4.60	<0.001 ***	27.8%
HEIGHT	−0.42	0.06	−0.54 × −0.31	−7.09	<0.001 ***	
ANGULATION	0.05	0.02	0.02 × 0.08	3.28	0.001 **	
U6D	0.04	0.35	−0.65 × 0.72	0.10	0.921	
U67	1.37	0.32	0.74 × 1.99	4.27	<0.001 ***	
U7M	−0.07	0.42	−0.90 × 0.76	−0.16	0.876	

** *p* < 0.01; *** *p* < 0.001.

**Table 10:** Results of meta-regression of bone thickness by facial pattern: beta coefficient, standard error (SE), 95% confidence interval, z test (*p*-value), and R^2^.

	Beta	SE	95% CI	z	* p * -Value	R^2^
intercept	3.11	0.39	2.36 3.87	8.06	<0.001 ***	0.0%
Brachy	0.16	0.54	−0.89 1.22	0.31	0.760	
Dolico	0.27	0.54	−0.78 1.33	0.51	0.611	

*** *p* < 0.001.

## Supplementary Materials

The following supporting information can be downloaded at: https://www.mdpi.com/article/10.3390/jcm14114005/s1: PRSIMA checklist; modified STROBE checklist for cross-sectional studies (results table of the data extracted from the final selected studies); meta-analysis results tables and figures; tables on the search results and exclusion reasons in the process of assessing the reports for eligibility.

## References

[1] Matias M., Flores-Mir C., Rodrigues de Almeida M., da Silva Vieira B., Salvatore de Freitas K.M., Calabrese Nunes D., Ferreira M.C., Ursi W. (2021). Miniscrew Insertion Sites of Infrazygomatic Crest and Mandibular Buccal Shelf in Different Vertical Craniofacial Patterns: A Cone-Beam Computed Tomography Study. Korean J. Orthod..

[2] Vargas E.O.A., Lopes de Lima R., Nojima L.I. (2020). Mandibular Buccal Shelf and Infrazygomatic Crest Thicknesses in Patients with Different Vertical Facial Heights. Am. J. Orthod. Dentofac. Orthop. Off. Publ. Am. Assoc. Orthod. Its Const. Soc. Am. Board Orthod..

[3] Murugesan A., Jain R.K. (2020). A 3D Comparison of Dimension of Infrazygomatic Crest Region in Different Vertical Skeletal Patterns: A Retrospective Study. Int. Orthod..

[4] Uribe F., Mehr R., Mathur A., Janakiraman N., Allareddy V. (2015). Failure Rates of Mini-Implants Placed in the Infrazygomatic Region. Prog. Orthod..

[5] Sanchis C.R., Pérez-Varela J.C., Zamora-Martínez N., García-Sanz V., Tarazona-Álvarez B., Paredes-Gallardo V. (2024). Optimal Sites for Mini-Implant Insertion into the Infrazygomatic Crest According to Different Craniofacial Morphologies: A Cross-Sectional Cone-Beam Computed Tomography Study. Am. J. Orthod. Dentofacial Orthop..

[6] He Y., Liu J., Huang R., Chen X., Jia X., Zeng N., Fan X., Huang X. (2023). Clinical Analysis of Successful Insertion of Orthodontic Mini-Implants in Infrazygomatic Crest. BMC Oral Health.

[7] Patil S.R., Ansari S., Nene S., Kalia A., Hegde A., Joshi J. (2024). To Evaluate the Cortical Bone Thickness for Ideal Placement of Infrazygomatic Crest (IZC) and Mandibular Buccal Shelf (MCS) Bone Screws in Patients with Different Facial Pattern Using Cone-Beam Computed Tomography (CBCT). J. Contemp. Orthod..

[8] Sreenivasagan S., Subramanian A.K., Chae J.M. (2024). Comparison of Treatment Effects during En-masse Retraction of Upper Anterior Teeth Placed Using Mini-implants Placed at Infrazygomatic Crest and Interradicular Sites: A Randomized Controlled Trial. Orthod. Craniofac. Res..

[9] Liu H., Wu X., Yang L., Ding Y. (2017). Safe Zones for Miniscrews in Maxillary Dentition Distalization Assessed with Cone-Beam Computed Tomography. Am. J. Orthod. Dentofacial Orthop..

[10] Sharan J., Bajoria A., Jena A.K., Sinha P., Shivakumar A., Kamal V.K., Marya A. (2024). Assessment of Bone Thickness at the Infra Zygomatic Crest Region for Various Orthodontic Miniscrew Implant (OMSI) Insertion Angles: A Cone-Beam Computed Tomographic Study. Turk. J. Orthod..

[11] Liou E.J.W., Chen P.-H., Wang Y.-C., Lin J.C.-Y. (2007). A Computed Tomographic Image Study on the Thickness of the Infrazygomatic Crest of the Maxilla and Its Clinical Implications for Miniscrew Insertion. Am. J. Orthod. Dentofacial Orthop..

[12] Wani M.A., Shukla D., Amir M., Siddiqui S., Mehtab S., Jafar M.S., Khan M.d.A.H., Rasool M. (2023). Infra Zygomatic Crest (IZC) and Mandibular Buccal Shelf (MBS) Bone Screws: A Comprehensive Updated Review. J. Dent. Spec..

[13] Patil S., Shaikh A., Galgali S.A., Jamdar A.F., Patel I. (2022). Efficacy of Infrazygomatic Crest Implants for Full-Arch Distalization of Maxilla and Reduction of Gummy Smile in Class II Malocclusion. J. Contemp. Dent. Pract..

[14] Sreenivasagan S., Subramanian A.K., Chae J.M., Venugopal A., Marya A. (2021). Displacement Patterns of the Maxillary Anterior Teeth during Total Distalization and En Masse Anterior Retraction Using Interradicular and Infrazygomatic Crest Mini-Implants with Varying Power Arm Heights: A Finite Element Analysis. J. Int. Oral Health.

[15] Gill G., Shashidhar K., Kuttappa M.N., Kushalappa P.B.D., Sivamurthy G., Mallick S. (2023). Failure Rates and Factors Associated with Infrazygomatic Crestal Orthodontic Implants—A Prospective Study. J. Oral Biol. Craniofacial Res..

[16] Arvind T.R.P., Jain R.K. (2021). Computed Tomography Assessment of Maxillary Bone Density for Orthodontic Mini-Implant Placement with Respect to Vertical Growth Patterns. J. Orthod..

[17] Krishnakumaran M., Krishnan B., Raman R., Rangarajan S., Preethi G., Chinnasamy A. (2022). Correlation of Infrazygomatic Bone Thickness with Cervical Vertebrae Maturation Stages. J. Indian Orthod. Soc..

[18] Chang C.H., Lin J.-H., Roberts W.E. (2022). Success of Infrazygomatic Crest Bone Screws: Patient Age, Insertion Angle, Sinus Penetration, and Terminal Insertion Torque. Am. J. Orthod. Dentofac. Orthop. Off. Publ. Am. Assoc. Orthod. Its Const. Soc. Am. Board Orthod..

[19] Shetty S., Ramesh A., Maniyankod S.B., Parveen K., Selvakumar S.G., Mubeen M., Amin V. (2024). Comparing the Efficiency of Infrazygomatic Crest (IZC) Screws and Conventional Method for Anterior Retraction in Patients Undergoing Fixed Orthodontic Treatment for Class 2 Malocclusion: A Prospective Clinical Study. Cureus.

[20] Jia X., Chen X., Huang X. (2018). Influence of Orthodontic Mini-Implant Penetration of the Maxillary Sinus in the Infrazygomatic Crest Region. Am. J. Orthod. Dentofac. Orthop. Off. Publ. Am. Assoc. Orthod. Its Const. Soc. Am. Board Orthod..

[21] Baghel S., Kujur A.R., Gopal B.V., Kumari J., Gupta A.K., Singh V.K. (2024). Evaluation of Bone Thickness at Infra-Zygomatic Crest Region Compared with Cervical Vertebrae Maturation Index. Bioinformation.

[22] Page M.J., McKenzie J.E., Bossuyt P.M., Boutron I., Hoffmann T.C., Mulrow C.D., Shamseer L., Tetzlaff J.M., Akl E.A., Brennan S.E. (2021). The PRISMA 2020 Statement: An Updated Guideline for Reporting Systematic Reviews. BMJ.

[23] Nelson S.J. (2019). Wheeler’s Dental Anatomy, Physiology and Occlusion—E-Book: Wheeler’s Dental Anatomy, Physiology and Occlusion—E-Book.

[24] Tang Y., Lu W., Zhang Y., Wu W., Sun Q., Zhang Y., Liu X., Liang W., Chen S., Han B. (2024). Variations in the Alveolar Bone Morphology in Maxillary Molar Area: A Retrospective CBCT Study. BMC Oral Health.

[25] Damang T., Mahasantipiya P., Suteerapongpun P., Tripuwabhrut K. (2022). A Computed Tomographic Image Study on Thickness of the Modified Infrazygomatic Crest Site Between Patients with Class I and Class III Skeletal Pattern. Chiang Mai Dent. J..

[26] Pan Y., Wei L., Zheng Z., Bi W. (2024). An Evaluation of Bone Depth at Different Three-Dimensional Paths in Infrazygomatic Crest Region for Miniscrew Insertion: A Cone Beam Computed Tomography Study. Heliyon.

[27] Pan Y., Zheng Z., Bi W. (2024). An Evaluation of Infrazygomatic Crest Bone Thickness in Adolescents at Different Eruption Stages of the Maxillary Second Molar Detected by Cone Beam CT. Fudan Univ. J. Med. Sci..

[28] Ujala Saif A.M.S., Sundus Shabir S.S., Komal Majeed R.N. (2022). Assessment of Infrazygomatic Bone Thickness for Safe Placement of Infrazygomatic Implant in Pakistani Population; A Cbct Study—Khyber Journal of Medical Sciences. Khyber J. Med. Sci..

[29] Du B., Zhu J., Li L., Fan T., Tan J., Li J. (2021). Bone Depth and Thickness of Different Infrazygomatic Crest Miniscrew Insertion Paths between the First and Second Maxillary Molars for Distal Tooth Movement: A 3-Dimensional Assessment. Am. J. Orthod. Dentofac. Orthop. Off. Publ. Am. Assoc. Orthod. Its Const. Soc. Am. Board Orthod..

[30] Murugesan A., Sivakumar A. (2020). Comparison of Bone Thickness in Infrazygomatic Crest Area at Various Miniscrew Insertion Angles in Dravidian Population—A Cone Beam Computed Tomography Study. Int. Orthod..

[31] Dangal R., Shrestha R.M., Dhakal J., Shrestha S., Gupta A., Shah S. (2022). Comparison of Bone Thickness in Infrazygomatic Crest Area at Various Miniscrew Insertion Angles: A Cone-Beam Computed Tomographic Study. Taiwan. J. Orthod..

[32] Balachandran H., Shafeequdheen P., Varghese S.T., Unni M.C., Suparna K. (2024). Evaluation of Bone Thickness at Different Anatomical Sites in Infrazygomatic Crest for Miniscrew Insertion in Skeletal Class II Patients—A CBCT Study. IOSR J. Dent. Med. Sci..

[33] Hariharno S., Goel S., Gupta N., Kumar A., Susar S. (2024). Optimal Placement Site and Angulation for Infrazygomatic Screw’s Insertion—A CBCT Study. Acad. J. Med..

[34] Gibas-Stanek M., Ślusarska J., Urzędowski M., Żabicki S., Pihut M. (2023). Quantitative Evaluation of the Infrazygomatic Crest Thickness in Polish Subjects: A Cone-Beam Computed Tomography Study. Appl. Sci..

[35] Lima A., Domingos R.G., Cunha Ribeiro A.N., Rino Neto J., De Paiva J.B. (2022). Safe Sites for Orthodontic Miniscrew Insertion in the Infrazygomatic Crest Area in Different Facial Types: A Tomographic Study. Am. J. Orthod. Dentofacial Orthop..

[36] Mathew S., Shivamurthy P.G., Kumar M.S., Kumar A. (2023). Three-Dimensional Comparison of Infra-Zygomatic Crest Thickness in Different Facial Patterns: A Cross-Sectional Study. World J. Dent..

[37] Tavares A., Montanha-Andrade K., Cury P.R., Crusoé-Rebello I., Neves F.S. (2022). Tomographic Assessment of Infrazygomatic Crest Bone Depth for Extra-alveolar Miniscrew Insertion in Subjects with Different Vertical and Sagittal Skeletal Patterns. Orthod. Craniofac. Res..

[38] Luchini C., Veronese N., Nottegar A., Shin J.I., Gentile G., Granziol U., Soysal P., Alexinschi O., Smith L., Solmi M. (2021). Assessing the Quality of Studies in Meta-Research: Review/Guidelines on the Most Important Quality Assessment Tools. Pharm. Stat..

[39] Husseini B., Younes R., Baumgaertel S., El Wak T., El Osta N., Bassil-Nassif N., Bouserhal J. (2022). Assessment of Infrazygomatic Crest Dimensions in Different Vertical Facial Growth Types for Miniscrew Insertion: A Cone-Beam Computed Tomography Study. Am. J. Orthod. Dentofac. Orthop. Off. Publ. Am. Assoc. Orthod. Its Const. Soc. Am. Board Orthod..

[40] Costea M.-C., Bondor C.-I., Muntean A., Badea M.E., Mesaroş A.-Ş., Kuijpers-Jagtman A.M. (2018). Proximity of the Roots of Posterior Teeth to the Maxillary Sinus in Different Facial Biotypes. Am. J. Orthod. Dentofacial Orthop..

