# Supplementary material for: Optimizing Infrazygomatic Miniscrew Insertion Parameters: Systematic Review and Meta-Regression Analysis of Bone Thickness by Insertion Height, Angulation, and Anatomical Position
Source: J Clin Med. 2025 Jun 5;14(11):4005. doi: 10.3390/jcm14114005 (PMC12156382; doi:10.3390/jcm14114005)
Supplement: Supplementary file 1 [file jcm-14-04005-s001.zip › jcm-3654906-suplementary.pdf]

Section and Topic	Item #	Checklist item	Location where item is reported							
			Mathew et al. 2023	Sharan et al., 2024	Hariharno et al. (2024)	Damang et al. (2022)	Ujala Saif et al. (2022)	Murugesan and Sivakumar (2020	Lima et al. (2022)	PAN Ying-dan et al. (2024)
**TITLE**										
Title	1	Identify the report as a systematic review.	NO: Title page	NO: Title page	NO	NO	NO	NO	NO	NO
**ABSTRACT**										
Abstract	2	See the PRISMA 2020 for Abstracts checklist.	YES: 552	YES: 104	YES 35	YES: 72	YES: 135	YES: 105	YES: 1	YES: 580
**INTRODUCTION**										
Rationale	3	Describe the rationale for the review in the context of existing knowledge.	YES: 553-554	YES: 105-106	YES: 36	YES: 73	YES: 135	YES: 105	YES: 2	YES: 580-581
Objectives	4	Provide an explicit statement of the objective(s) or question(s) the review addresses.	YES: 553	YES: 105	YES: 36	YES: 73	YES: 135	YES: 105	YES: 2	YES: 581
**METHODS**										
Eligibility criteria	5	Specify the inclusion and exclusion criteria for the review and how studies were grouped for the syntheses.	YES: 553	YES: 105	YES: 36-37	YES: 73	YES: 136	YES: 107	YES: 3	YES: 582
Information sources	6	Specify all databases, registers, websites, organisations, reference lists and other sources searched or consulted to identify studies. Specify the date when each source was last searched or consulted.	YES: 553	YES: 105	YES: 36-37	YES: 73	YES: 136	YES: 107	YES: 3	YES: 582
Search strategy	7	Present the full search strategies for all databases, registers and websites, including any filters and limits used.	NO	NO	NO	NO	NO	NO	NO	NO
Selection process	8	Specify the methods used to decide whether a study met the inclusion criteria of the review, including how many reviewers screened each record and each report retrieved, whether they worked independently, and if applicable, details of automation tools used in the process.	NO	YES: 105	NO	NO	NO	NO	NO	NO
Data collection process	9	Specify the methods used to collect data from reports, including how many reviewers collected data from each report, whether they worked independently, any processes for obtaining or confirming data from study investigators, and if applicable, details of automation tools used in the process.	YES: 553-554	YES: 106-107	YES: 36-37	YES: 73-74	YES: 136-137	YES: 107	YES: 3	YES: 582-583
Data items	10a	List and define all outcomes for which data were sought. Specify whether all results that were compatible with each outcome domain in each study were sought (e.g. for all measures, time points, analyses), and if not, the methods used to decide	YES: 553	YES: 106	YES: 36-37	YES: 73-74	YES: 136-137	YES: 107	YES: 4	YES: 583

Section and Topic	Item #	Checklist item	Location where item is reported							
		which results to collect.								
	10b	List and define all other variables for which data were sought (e.g. participant and intervention characteristics, funding sources). Describe any assumptions made about any missing or unclear information.	NO	NO	YES: 37	YES: 73-74	YES: 136-137	YES: 107	YES: 4	YES: 583
Study risk of bias assessment	11	Specify the methods used to assess risk of bias in the included studies, including details of the tool(s) used, how many reviewers assessed each study and whether they worked independently, and if applicable, details of automation tools used in the process.	NO	NO	NO	NO	NO	NO	NO	NO
Effect measures	12	Specify for each outcome the effect measure(s) (e.g. risk ratio, mean difference) used in the synthesis or presentation of results.	YES: 553	YES: 106	YES: 37-38	YES: 75	YES: 137	YES: 4	YES: 4	YES: 583-584
Synthesis methods	13a	Describe the processes used to decide which studies were eligible for each synthesis (e.g. tabulating the study intervention characteristics and comparing against the planned groups for each synthesis (item #5)).	NO	NO	NO	NO	NO	NO	NO	NO
	13b	Describe any methods required to prepare the data for presentation or synthesis, such as handling of missing summary statistics, or data conversions.	NO	NO	NO	NO	NO	NO	NO	NO
	13c	Describe any methods used to tabulate or visually display results of individual studies and syntheses.	YES: 556-558	YES: 107-109	YES: 38-39-40	YES: 74-75	YES: 137-138	YES: 107-108	YES: 5-6	YES: 584-585
	13d	Describe any methods used to synthesize results and provide a rationale for the choice(s). If meta-analysis was performed, describe the model(s), method(s) to identify the presence and extent of statistical heterogeneity, and software package(s) used.	NO	NO	NO	NO	NO	NO	YES: 6	585
	13e	Describe any methods used to explore possible causes of heterogeneity among study results (e.g. subgroup analysis, meta-regression).	NO	NO	NO	NO	NO	NO	NO	NO
	13f	Describe any sensitivity analyses conducted to assess robustness of the synthesized results.	NO	NO	NO	NO	NO	NO	NO	NO
Reporting bias assessment	14	Describe any methods used to assess risk of bias due to missing results in a synthesis (arising from reporting biases).	NO	NO	NO	NO	NO	NO	NO	NO
Certainty assessment	15	Describe any methods used to assess certainty (or confidence) in the body of evidence for an outcome.	NO	NO	NO	NO	NO	NO	NO	NO
**RESULTS**										
Study selection	16a	Describe the results of the search and selection process, from the number of records identified in the search to the number of studies included in the review, ideally using a flow diagram.	YES: 553-554	YES: 105	YES: 37	YES: 73-74	NO	NO	NO	NO
	16b	Cite studies that might appear to meet the inclusion criteria, but which were excluded, and explain why	NO	NO	NO	NO	NO	NO	NO	NO

Section and Topic	Item #	Checklist item	Location where item is reported							
		they were excluded.								
Study characteristics	17	Cite each included study and present its characteristics.	YES: 554	YES: 106	YES: 37-38	YES: 73	NO	NO	YES: 3	YES: 582-583
Risk of bias in studies	18	Present assessments of risk of bias for each included study.	NO	NO	NO	NO	NO	NO	NO	NO
Results of individual studies	19	For all outcomes, present, for each study: (a) summary statistics for each group (where appropriate) and (b) an effect estimate and its precision (e.g. confidence/credible interval), ideally using structured tables or plots.	NO	YES: 107-109	YES: 38-39-40	YES: Page 75	YES: 137-138	YES: 108-109-110-111	YES: 5-6	YES: 583-584-585
Results of syntheses	20a	For each synthesis, briefly summarise the characteristics and risk of bias among contributing studies.	NO	NO	NO	NO	NO	NO	NO	NO
	20b	Present results of all statistical syntheses conducted. If meta-analysis was done, present for each the summary estimate and its precision (e.g. confidence/credible interval) and measures of statistical heterogeneity. If comparing groups, describe the direction of the effect.	NO	NO	YES: 38-39-40	YES: 74-75-76	YES: 137-138	YES: 108-109-110-111	YES: 6-7	YES: 583-584-585
	20c	Present results of all investigations of possible causes of heterogeneity among study results.	NO	NO	NO	NO	NO	NO	NO	NO
	20d	Present results of all sensitivity analyses conducted to assess the robustness of the synthesized results.	NO	NO	NO	NO	NO	NO	NO	NO
Reporting biases	21	Present assessments of risk of bias due to missing results (arising from reporting biases) for each synthesis assessed.	NO	NO	NO	NO	NO	NO	NO	NO
Certainty of evidence	22	Present assessments of certainty (or confidence) in the body of evidence for each outcome assessed.	NO	NO	NO	NO	NO	NO	NO	NO
**DISCUSSION**										
Discussion	23a	Provide a general interpretation of the results in the context of other evidence.	YES: 557-558	YES: 109	YES: 37-38	YES: 76	YES: 138-139	YES: 110-111	YES: 7	YES: 583-584-585
	23b	Discuss any limitations of the evidence included in the review.	YES: 557	YES: 109	NO	YES: 76	YES: 138-139	YES: 113	NO	NO
	23c	Discuss any limitations of the review processes used.	YES: 557	YES: 109	NO	YES: 76	YES: 138-139	YES: 113	NO	NO
	23d	Discuss implications of the results for practice, policy, and future research.	YES: 558	YES: 109	YES: 40-41	YES: 76	YES: 138-139	YES: 113	YES: 7-8	YES: 587
**OTHER INFORMATION**										
Registration and protocol	24a	Provide registration information for the review, including register name and registration number, or state that the review was not registered.	NO	NO	NO	NO	NO	NO	NO	YES: 586
	24b	Indicate where the review protocol can be accessed, or state that a protocol was not prepared.	NO	NO	NO	NO	NO	NO	NO	NO

Section and Topic	Item #	Checklist item	Location where item is reported							
	24c	Describe and explain any amendments to information provided at registration or in the protocol.	NO	NO	NO	NO	NO	NO	NO	NO
Support	25	Describe sources of financial or non-financial support for the review, and the role of the funders or sponsors in the review.	YES: 552	YES: 104	NO	YES: 77	YES: 139	NO	YES:8	YES: 580
Competing interests	26	Declare any competing interests of review authors.	YES: 558	YES: 109	NO	NO	NO	NO	NO	NO
Availability of data, code and other materials	27	Report which of the following are publicly available and where they can be found: template data collection forms; data extracted from included studies; data used for all analyses; analytic code; any other materials used in the review.	NO	NO	NO	NO	NO	NO	NO	NO

From: Page MJ, McKenzie JE, Bossuyt PM, Boutron I, Hoffmann TC, Mulrow CD, et al. The PRISMA 2020 statement: an updated guideline for reporting systematic reviews. BMJ 2021;372:n71. doi: 10.1136/bmj.n71

For more information, visit: http://www.prisma-statement.org/

Section and Topic	Item #	Checklist item	Location where item is reported								
			Gibas-Stanek et al. (2023)	Balachandran et al. (2024)	Liou et al. (2007)	Du et al., 2021	Tavares et al., 2022	Matias et al., 2021	Yingdan Pan et al. (2024)	Dangal et al. (2022)	Sanchis et al. (2024)
**TITLE**											
Title	1	Identify the report as a systematic review.	NO: Title page	NO: Title page	NO	NO: Title page	NO: Title page	NO: Title page	NO: Title page	NO: Title page	NO: Title page
**ABSTRACT**											
Abstract	2	See the PRISMA 2020 for Abstracts checklist.	YES: 1	YES: 1	YES: 352	YES: 113	YES: 49	YES: 387	YES: 1	YES: 185	YES: 1
**INTRODUCTION**											
Rationale	3	Describe the rationale for the review in the context of existing knowledge.	YES: 2	YES: 2	YES: 352	YES: 113-114	YES: 49-50	YES: 387-388	YES: 2	YES: 185	YES: 2

Section and Topic	Item #	Checklist item	Location where item is reported								
Objectives	4	Provide an explicit statement of the objective(s) or question(s) the review addresses.	YES: 2	YES: 1	YES: 352	YES: 113	YES: 49	YES: 387	YES: 2	YES: 185	YES: 2
**METHODS**											
Eligibility criteria	5	Specify the inclusion and exclusion criteria for the review and how studies were grouped for the syntheses.	YES: 3	YES: 2	YES: 353	YES: 114	YES: 50	YES: 388	YES: 3	YES: 186	YES: 2-3
Information sources	6	Specify all databases, registers, websites, organisations, reference lists and other sources searched or consulted to identify studies. Specify the date when each source was last searched or consulted.	YES: 3	YES: 2	YES: 353	YES: 114	YES: 50	YES: 388	YES: 3	YES: 186	YES: 2-3
Search strategy	7	Present the full search strategies for all databases, registers and websites, including any filters and limits used.	NO	NO	NO	NO	NO	NO	NO	NO	NO
Selection process	8	Specify the methods used to decide whether a study met the inclusion criteria of the review, including how many reviewers screened each record and each report retrieved, whether they worked independently, and if applicable, details of automation tools used in the process.	NO	NO	NO	YES: 114	YES: 50	YES: 388	NO	NO	NO
Data collection process	9	Specify the methods used to collect data from reports, including how many reviewers collected data from each report, whether they worked independently, any processes for obtaining or confirming data from study investigators, and if applicable, details of automation tools used in the process.	YES: 4	YES: 2-3-4	YES: 353	YES: 114-115	YES: 50-51	YES: 388-389	YES: 4	YES: 188	YES: 2-3-4
Data items	10a	List and define all outcomes for which data were sought. Specify whether all results that were compatible with each outcome domain in each study were sought (e.g. for all measures, time points, analyses), and if not, the methods used to decide which results to collect.	YES: 5	YES: 4	YES: 353	YES: 114	YES: 50-51	YES: 389	YES: 5	YES: 188	YES: 5-6
	10b	List and define all other variables for which data were sought (e.g. participant and intervention characteristics, funding sources). Describe any assumptions made	YES: 5	YES: 4	YES: 353	NO	NO	NO	YES: 5	YES: 188	YES: 5-6

Section and Topic	Item #	Checklist item	Location where item is reported								
		about any missing or unclear information.									
Study risk of bias assessment	11	Specify the methods used to assess risk of bias in the included studies, including details of the tool(s) used, how many reviewers assessed each study and whether they worked independently, and if applicable, details of automation tools used in the process.	YES: 4	NO	NO	NO	NO	NO	NO	NO	YES: 6
Effect measures	12	Specify for each outcome the effect measure(s) (e.g. risk ratio, mean difference) used in the synthesis or presentation of results.	YES: 6	YES: 4	YES: 354	YES: 114	YES: 50	YES: 389	YES: 6	YES: 188	YES: 6
Synthesis methods	13a	Describe the processes used to decide which studies were eligible for each synthesis (e.g. tabulating the study intervention characteristics and comparing against the planned groups for each synthesis (item #5)).	NO	NO	NO	NO	NO	NO	NO	NO	NO
	13b	Describe any methods required to prepare the data for presentation or synthesis, such as handling of missing summary statistics, or data conversions.	NO	NO	NO	NO	NO	NO	NO	NO	NO
	13c	Describe any methods used to tabulate or visually display results of individual studies and syntheses.	NO	NO	YES: 354-355	YES: 116-118	YES: 52-53	YES: 391-392	NO	NO	NO
	13d	Describe any methods used to synthesize results and provide a rationale for the choice(s). If meta-analysis was performed, describe the model(s), method(s) to identify the presence and extent of statistical heterogeneity, and software package(s) used.	NO	NO	NO	NO	NO	NO	NO	NO	NO
	13e	Describe any methods used to explore possible causes of heterogeneity among study results (e.g. subgroup analysis, meta-regression).	NO	NO	NO	NO	NO	NO	NO	NO	NO
	13f	Describe any sensitivity analyses conducted to assess robustness of the synthesized results.	YES:8	NO	NO	NO	NO	NO	NO	NO	YES:10
Reporting bias assessment	14	Describe any methods used to assess risk of bias due to missing results in a synthesis (arising from reporting biases).	NO	NO	NO	NO	NO	NO	NO	NO	NO
Certainty assessment	15	Describe any methods used to assess certainty (or confidence) in the body of evidence for an outcome.	YES: 8	NO	NO	NO	NO	NO	NO	NO	NO
**RESULTS**											
Study selection	16a	Describe the results of the search and	YES: 4	YES: 4	YES:	YES: 114	YES: 50	YES: 388	YES: 4	YES:	YES: 6

Section and Topic	Item #	Checklist item	Location where item is reported								
		selection process, from the number of records identified in the search to the number of studies included in the review, ideally using a flow diagram.			353					187	
	16b	Cite studies that might appear to meet the inclusion criteria, but which were excluded, and explain why they were excluded.	NO	NO	NO	NO	NO	NO	NO	NO	NO
Study characteristics	17	Cite each included study and present its characteristics.	YES: 5	YES: 4-5	YES: 353	YES: 114	YES: 50	YES: 388	YES: 5	YES: 188-189	YES: 5-6
Risk of bias in studies	18	Present assessments of risk of bias for each included study.	NO	NO	NO	NO	NO	NO	NO	NO	NO
Results of individual studies	19	For all outcomes, present, for each study: (a) summary statistics for each group (where appropriate) and (b) an effect estimate and its precision (e.g. confidence/credible interval), ideally using structured tables or plots.	YES: 7	YES: 6	YES: 354-355	YES: 116-118	YES: 52-53	YES: 391-392	YES: 7	YES: 189	YES: 7-8-9
Results of syntheses	20a	For each synthesis, briefly summarise the characteristics and risk of bias among contributing studies.	NO	NO	NO	NO	NO	NO	NO	NO	NO
	20b	Present results of all statistical syntheses conducted. If meta-analysis was done, present for each the summary estimate and its precision (e.g. confidence/credible interval) and measures of statistical heterogeneity. If comparing groups, describe the direction of the effect.	YES: 4-5-6-7-8	NO	YES: 354-355	NO	NO	NO	NO	YES: 188	YES: 7-8-9
	20c	Present results of all investigations of possible causes of heterogeneity among study results.	YES: 8	NO	NO	NO	NO	NO	NO	NO	YES: 10
	20d	Present results of all sensitivity analyses conducted to assess the robustness of the synthesized results.	YES: 8	NO	NO	NO	NO	NO	NO	NO	YES:10
Reporting biases	21	Present assessments of risk of bias due to missing results (arising from reporting biases) for each synthesis assessed.	NO	NO	NO	NO	NO	NO	NO	NO	NO
Certainty of evidence	22	Present assessments of certainty (or confidence) in the body of evidence for each outcome assessed.	NO	NO	NO	NO	NO	NO	NO	NO	NO
**DISCUSSION**											
Discussion	23a	Provide a general interpretation of the results in the context of other evidence.	NO	NO	YES: 355-356	YES: 118	YES: 53	YES: 392	NO	NO	NO
	23b	Discuss any limitations of the evidence included in the review.	YES: 11	NO	NO	YES: 118	YES: 53	YES: 392	YES: 9	NO	YES: 11
	23c	Discuss any limitations of the review	YES: 11	NO	NO	YES: 118	YES: 53	YES: 392	YES: 9	NO	YES: 11

Section and Topic	Item #	Checklist item	Location where item is reported								
		processes used.									
	23d	Discuss implications of the results for practice, policy, and future research.	YES: 11	YES: 6	YES: 356	YES: 118	YES: 53	YES: 392	YES: 9	YES: 188-189	YES: 11
**OTHER INFORMATION**											
Registration and protocol	24a	Provide registration information for the review, including register name and registration number, or state that the review was not registered.	YES: 11	YES: 6	NO	NO	NO	NO	YES: 9-10	YES: 189	YES: 1
	24b	Indicate where the review protocol can be accessed, or state that a protocol was not prepared.	YES: 11	NO	NO	NO	NO	NO	YES: 9	NO	YES: 1
	24c	Describe and explain any amendments to information provided at registration or in the protocol.	NO	NO	NO	NO	NO	NO	NO	NO	NO
Support	25	Describe sources of financial or non-financial support for the review, and the role of the funders or sponsors in the review.	NO	NO	NO	YES: 113	NO	NO	YES:9	YES: 189	NO
Competing interests	26	Declare any competing interests of review authors.	NO	NO	NO	YES: 118	YES: 53	YES: 392	NO	NO	NO
Availability of data, code and other materials	27	Report which of the following are publicly available and where they can be found: template data collection forms; data extracted from included studies; data used for all analyses; analytic code; any other materials used in the review.	YES: 11	YES: 7	NO	NO	NO	NO	YES: 10	YES: 189	YES: 11

From: Page MJ, McKenzie JE, Bossuyt PM, Boutron I, Hoffmann TC, Mulrow CD, et al. The PRISMA 2020 statement: an updated guideline for reporting systematic reviews. BMJ 2021;372:n71. doi: 10.1136/bmj.n71

For more information, visit: http://www.prisma-statement.org/

STROBE Checklist for cross-sectional studies (1)

STROBE Item	*Mathew et al. (2023)*	*Sharan et al. (2024)*	*Du et al. (2021)*	*Tavares et al. (2022)*	*Matias et al. (2021)*
**Title & Abstract**	Clearly states study design and key findings.	Clearly states study design and findings on IZC thickness.	Clearly states study objective and results related to bone depth and thickness.	Clearly states study objective, methodology, and key findings on bone depth at the IZC.	Clearly states study objective and findings on IZC and MBS miniscrew insertion.
**Background/Rationale**	Justifies evaluation of IZC thickness in different facial patterns.	Justifies importance of IZC thickness for miniscrew placement.	Justifies evaluation of IZC bone depth and thickness for optimal insertion paths.	Justifies the need to evaluate IZC bone depth variations in different skeletal patterns.	Justifies the need to evaluate IZC and MBS bone thickness in different craniofacial patterns for miniscrew placement.
**Objectives**	Aims to determine variations in IZC thickness and optimal implant placement.	Evaluates IZC bone thickness to guide miniscrew placement.	Aims to assess bone depth and thickness across different insertion paths.	Aims to determine bone availability at the IZC for miniscrew insertion.	Aims to identify optimal miniscrew insertion sites in IZC and MBS for different vertical craniofacial patterns.
**Study Design**	Identified as a cross-sectional CBCT study.	Cross-sectional CBCT-based study.	Cross-sectional study using CBCT and 3D reconstruction.	Cross-sectional study using multislice computed tomography (MSCT).	Cross-sectional study using CBCT reconstructions of untreated individuals.

**Setting**	Conducted in an academic dental setting.	Conducted in a hospital orthodontic department.	Conducted in a university hospital setting.	Conducted in a dental school setting.	Conducted at Guarulhos University, Brazil, using CBCT scans from a private practice.
**Participants**	Inclusion criteria clearly defined.	50 CBCT scans analyzed.	36 orthodontic patients analyzed.	58 adult patients from an image bank analyzed.	45 untreated individuals classified as brachyfacial, mesofacial, or dolichofacial, based on craniofacial pattern.
**Variables**	IZC thickness and insertion height measured.	IZC thickness and insertion angles analyzed.	Bone thickness and depth were primary variables.	Bone depth measured at different insertion angles and heights.	Bone thickness at the IZC and MBS measured at different heights and locations.
**Data Sources & Measurement**	CBCT imaging used with standardized protocols.	CBCT imaging analyzed using reference planes.	CBCT imaging and 3D reconstructions used.	MSCT images analyzed using OsiriX software.	CBCT images analyzed using Dolphin software with 3D reconstructions.
**Bias**	Efforts to minimize bias include standardized imaging protocols.	Bias controlled using strict inclusion criteria.	Bias reduced through controlled imaging conditions.	Bias reduced by using a standardized head position.	Bias minimized through standardized CBCT acquisition protocols and single examiner measurements.
**Study Size**	Sample size determined statistically.	Sample size calculated with power analysis.	Sample size based on statistical considerations.	Sample size determined based on confidence level.	Sample size determined based on power analysis (alpha 0.05, beta 0.2).
**Quantitative Variables**	Bone thickness and insertion height analyzed.	Bone thickness across different insertion angles.	Bone depth and thickness analyzed at different insertion paths.	Bone depth at different angles and distances measured.	Bone thickness at IZC and MBS analyzed at different distances from anatomical landmarks.
**Statistical Methods**	ANOVA and t-tests used.	t-tests and descriptive statistics applied.	Wilcoxon signed-rank tests used.	ANOVA and Tukey's post hoc test applied.	ANOVA and Tukey test used for intergroup comparisons.
**Results - Participants**	Reports subject distribution.	Reports participant distribution.	Reports participant demographics.	Reports participant demographics and skeletal classifications.	Reports distribution of participants among craniofacial patterns with age and gender details.

**Results - Descriptive Data**	Provides demographic and anatomical data.	Reports bone thickness variations.	Provides bone depth variations.	Reports bone depth differences by skeletal classification.	Provides descriptive bone thickness values at the IZC and MBS for each craniofacial group.
**Results - Outcome Data**	Reports IZC thickness and insertion height.	Reports IZC bone thickness.	Reports bone depth and thickness at different paths.	Reports bone depth changes at different insertion sites.	Reports IZC and MBS thickness at different insertion heights.
**Results - Main Results**	Horizontal growers have thicker IZC crestal bone.	IZC bone thickness increases with higher angles.	Negative correlation between bone depth and thickness.	Bone availability is lower in certain skeletal patterns.	No significant differences in IZC thickness between groups; MBS thickness varies between facial types.
**Results - Other Analyses**	Additional comparisons between measurement sites.	Compares right vs. left side thickness.	Compares bone depth and thickness across paths.	Analyzes bone depth across skeletal classifications.	Compares IZC and MBS thickness variations based on craniofacial type.
**Discussion - Key Results**	Supports IZC for mini-implant placement.	Suggests optimal miniscrew insertion angles.	Highlights bone depth variations for safe insertion.	Emphasizes importance of insertion angle selection.	Suggests craniofacial pattern influences MBS bone thickness but has minimal effect on IZC thickness.
**Discussion - Limitations**	Acknowledges lack of gender-specific analysis.	Acknowledges morphological variations in IZC thickness.	Notes imaging accuracy and sample diversity limitations.	Recognizes small sample size and reliance on retrospective data.	Acknowledges small sample size and difficulty excluding borderline facial types.
**Discussion – Interpretation**	Results interpreted with reference to clinical applications.	Results compared with IZC miniscrew placement studies.	Interpreted in orthodontic biomechanical context.	Discusses anatomical safety for IZC miniscrews.	Results interpreted in the context of CBCT evaluation for ideal insertion locations.
**Discussion - Generalisability**	Findings applicable but require validation.	Results applicable but require broader population validation.	Findings may not be generalizable across all skeletal patterns.	Results may not be generalizable due to sample selection.	Results applicable to Brazilian population but may not generalize to all ethnic groups.
**Other Information - Funding**	No external funding reported.	No external funding reported.	Funded by the National Natural	Funding information not provided.	No external funding reported.

			Science Foundation of China.		
**Overall Compliance with STROBE**	High	High	High	High	High
**Comments**	Aligns well with STROBE guidelines.	Strong methodology and statistical validation.	Well-structured methodology with robust 3D analysis.	Clear statistical validation and clinical relevance.	Well-designed study with clear clinical applicability.

STROBE Checklist for cross-sectional studies (2)

**STROBE Item**	***Sanchis et al. (2024)***	***Yingdan Pan et al. (2024)***	***Dangal et al. (2022)***	***Gibas-Stanek et al. (2023)***	***Balachandran et al. (2024)***
**Title & Abstract**	Clearly states study design and key findings related to optimal IZC mini-implant sites.	Clearly describes study design and findings on bone depth at different 3D paths in the IZC region.	Clearly mentions study objective and findings on IZC bone thickness and miniscrew insertion angles.	Clearly states study objective and its significance in Polish population.	Clearly states study objective focusing on IZC bone thickness in Class II patients.
**Background/Rationale**	Justifies need to examine IZC depth for safe mini-implant placement and its correlation	Justifies assessment of IZC bone depth at different insertion angles	Highlights the importance of evaluating IZC thickness for miniscrew	Justifies need to evaluate IZC bone thickness in a regional sample.	Justifies the need to examine IZC bone thickness at different anatomical sites.

	with skeletal classification.	to optimize placement.	stability and safety.		
**Objectives**	Aims to quantify IZC depth and evaluate correlations with skeletal classification, age, and gender.	Evaluates bone depth at different 3D paths to optimize insertion trajectory.	Determines IZC bone thickness, height, and optimal insertion angles.	Examines IZC thickness at different insertion points and its correlation with age and sex.	Assesses bone thickness at various anatomical points in skeletal Class II patients.
**Study Design**	Cross-sectional CBCT study.	Cross-sectional CBCT study.	Cross-sectional CBCT study.	Cross-sectional CBCT study.	Cross-sectional CBCT study.
**Setting**	Conducted at University of Valencia and private dental clinic (Spain).	Conducted at Zhongshan Hospital (Xiamen), Fudan University (China).	Conducted at Kantipur Dental College, Kathmandu (Nepal).	Conducted at University Dental Clinic in Krakow, Poland.	Conducted at PSM Dental College, Kerala, India.
**Participants**	201 CBCT scans of adults (20-40 years, no orthodontic history).	80 CBCT scans of adults (19-38 years, no missing or supernumerary teeth).	44 CBCT scans of adults (>18 years, no craniofacial anomalies).	100 CBCT scans (50 males, 50 females, aged >12 years).	23 CBCT scans of skeletal Class II patients (>19 years).
**Variables**	IZC depth measured at different molar regions; correlations with skeletal classification, vertical pattern, age, and gender analyzed.	Bone depth measured at different insertion paths, heights, and inclinations; bone deficiency ratio calculated.	Bone thickness measured at different insertion angles (40°-75°) and heights (11-17 mm from occlusal plane).	IZC thickness measured at multiple points between first and second molars.	IZC bone thickness measured at multiple interdental and root sites.
**Data Sources & Measurement**	CBCT imaging analyzed using Carestream 3D software.	CBCT analyzed using SIDEXIS 4 Imaging Software.	CBCT analyzed using CS Imaging Suite.	CBCT imaging analyzed using InViVo Dental Viewer.	CBCT images analyzed using MicroDicom software.

**Bias**	Bias minimized through standardized CBCT protocols and single examiner measurements.	Efforts to minimize bias through standardized measurement methods.	Bias reduced by standardizing CBCT imaging and measurement techniques.	Bias minimized by a single trained examiner and standardized measurement protocols.	Bias minimized using standardized imaging protocols and measurement techniques.
**Study Size**	Sample size (n=201) determined based on power analysis.	Sample size (n=80) determined with statistical considerations.	Sample size (n=44) determined based on study feasibility.	Sample size (n=100) determined based on statistical power analysis.	Sample size (n=23) based on available cases and study feasibility.
**Quantitative Variables**	IZC depth measured at multiple molar locations; comparisons across skeletal patterns.	Bone depth analyzed at 81 insertion paths with varying angles.	Bone thickness measured at different angles and heights.	Bone thickness evaluated at different heights and anatomical locations.	Bone thickness measured at different anatomical sites and heights.
**Statistical Methods**	ANOVA, Bonferroni correction, and Spearman correlation applied.	Adjusted Friedman test, Wilcoxon signed-rank test, and Bonferroni correction used.	Student t-test used for comparisons of bone thickness and insertion height.	Descriptive statistics applied with correlation analysis.	ANOVA and Tukey’s post hoc tests used for statistical comparisons.
**Results - Participants**	Reports distribution by skeletal classification and vertical pattern.	Reports distribution by insertion angles and paths.	Reports variations in bone thickness across angles and heights.	Reports variations in IZC thickness between different patient groups.	Reports differences in IZC thickness at various insertion points.
**Results - Descriptive Data**	Provides bone depth measurements by skeletal class, gender, and vertical pattern.	Reports bone depth variations at different paths, heights, and inclinations.	Reports bone thickness variations across insertion angles and heights.	Provides bone thickness measurements by age and sex.	Provides comparative bone thickness data across insertion heights.

**Results - Outcome Data**	Identifies optimal IZC insertion points based on anatomical measurements.	Identifies optimal bone depth regions for miniscrew placement.	Finds optimal insertion site at 70° and 13 mm from occlusal plane.	Identifies optimal IZC regions for miniscrew placement in Polish subjects.	Finds safe insertion zones at 11 mm from CEJ and 70° inclination.
**Results - Main Results**	Normodivergent patients exhibited lowest IZC ridge height; mesiobuccal root region had greatest total length.	Maximum bone depth found 13 mm above occlusal plane at mesiobuccal root of maxillary second molar.	Bone thickness increases with greater insertion angle but decreases with height.	Bone thickness is greatest at interdental space between first and second molars.	Bone thickness is greater in interdental areas compared to root regions.
**Results - Other Analyses**	Compares differences between right and left side IZC dimensions.	Compares bone deficiency ratios at different insertion paths.	Compares bone thickness variations by insertion angles.	Compares bone thickness differences by age and sex.	Compares bone thickness variations across anatomical locations.
**Discussion - Key Results**	Supports IZC as a safe mini-implant site but highlights anatomical variations by skeletal class.	Suggests optimal insertion sites for safe and stable miniscrew placement.	Recommends optimal angle and height for mini-implant stability.	Suggests optimal IZC sites based on anatomical variations.	Suggests optimal insertion sites for miniscrews in Class II patients.
**Discussion - Limitations**	Acknowledges sample limitations and need for additional validation.	Recognizes potential measurement variability in CBCT analysis.	Notes small sample size and limited population diversity.	Acknowledges limitations in sample representativeness.	Recognizes limitations due to small sample size and single-center study.
**Discussion - Interpretation**	Results interpreted in clinical context of IZC miniscrew placement.	Results discussed in relation to insertion path optimization for stability.	Results linked to clinical application of miniscrew insertion angles.	Results interpreted within the context of Polish population.	Findings discussed within the context of Class II skeletal patterns.
**Discussion - Generalizability**	Findings applicable but	Results generalizable to similar	Results specific to the studied population and	Findings may not be generalizable to	Results applicable to Class II patients but

	require broader validation.	populations but require further research.	may not be widely generalizable.	other ethnic populations.	require validation in other populations.
**Other Information – Funding**	No external funding reported.	No external funding reported.	No external funding reported.	No external funding reported.	No external funding reported.
**Overall Compliance with STROBE**	High	High	High	High	High
**Comments**	Well-structured study with robust statistical validation.	Innovative 3D analysis for optimizing IZC insertion paths.	Provides clear insights into IZC miniscrew placement strategies.	Strong study with a focus on anatomical variations in a specific population.	Important study for understanding IZC characteristics in Class II patients.

STROBE Checklist for cross-sectional studies (3)

STROBE Item	*Liou et al. (2007)*	*Hariharno et al. (2024)*	*Damang et al. (2022)*	*Ujala Saif et al. (2022)*	*Murugesan & Sivakumar (2020)*
**Title & Abstract**	Clearly states study objective and key findings on IZC thickness and miniscrew insertion angles.	Clearly describes study objective and key findings on optimal IZC screw placement site and angulation.	Clearly states study objective and findings on IZC thickness in Class I and Class III patients.	Clearly states study objective and findings on IZC bone thickness in the Pakistani population.	Clearly states study objective and key findings on IZC bone thickness in the Dravidian population.
**Background/Rationale**	Justifies need for IZC thickness evaluation to determine safe miniscrew placement sites.	Justifies need to determine the safest insertion angle for IZC screws.	Highlights need to compare IZC thickness between skeletal patterns.	Justifies need for IZC thickness assessment to determine safe implant placement.	Justifies need to examine IZC thickness at different insertion angles.
**Objectives**	Measures IZC thickness at various angles and positions to determine safe insertion locations.	Evaluates IZC thickness and height at different insertion angles in an Indian population.	Compares IZC thickness and insertion angles in Class I vs. Class III patients.	Evaluates IZC bone thickness at different insertion angles.	Assesses bone thickness at various insertion angles in Dravidian adults.
**Study Design**	Computed tomography (CT) study.	Cross-sectional CBCT study.	Cross-sectional CBCT study.	Cross-sectional CBCT study.	Cross-sectional CBCT study.
**Setting**	Conducted at Chang Gung Memorial Hospital, Taipei, Taiwan.	Conducted at Maitri College of Dentistry and Research Centre, India.	Conducted at Chiang Mai University, Thailand.	Conducted at Khyber College of Dentistry, Peshawar, Pakistan.	Conducted at Saveetha University, Chennai, India.
**Participants**	16 adult patients (6 women, 10 men, mean age 27 years).	30 adult patients (15 males, 15 females, >18 years).	30 CBCT scans (15 Class I, 15 Class III).	116 CBCT scans (age >16 years).	10 CBCT scans (6 females, 4 males, 20-30 years).
**Variables**	IZC thickness measured at 40°-75° angles and 13-17 mm above occlusal plane.	IZC thickness measured at angles ranging from 40° to 75°.	IZC thickness measured at 55°-70° insertion angles and multiple vertical levels.	IZC thickness measured at 55°-75° insertion angles.	IZC thickness measured at angles ranging from 40° to 75°.

**Data Sources & Measurement**	CT images analyzed using Analyze (Mayo Clinic) software.	CBCT images analyzed using Theia imaging software.	CBCT images analyzed using Dolphin Imaging software.	CBCT images analyzed using SPSS for statistical analysis.	CBCT images analyzed using Galileos Viewer software.
**Bias**	Bias minimized by standardizing CT measurement protocols.	Efforts to reduce bias through standardized imaging techniques.	Bias minimized through standardized image acquisition.	Bias minimized through standardized imaging protocols.	Efforts to reduce bias through standardized measurement protocols.
**Study Size**	Sample size (n=16) based on hospital records; no formal power calculation.	Sample size (n=30) based on study feasibility; no formal power analysis.	Sample size (n=30) based on availability; no formal power calculation.	Sample size (n=116) determined using OpenEpi for power calculation.	Sample size (n=10) limited due to availability.
**Quantitative Variables**	Bone thickness measured at different angles and heights.	Bone thickness analyzed at various insertion angles.	Bone thickness compared across skeletal classes.	Bone thickness compared across insertion angles.	Bone thickness compared across different angles.
**Statistical Methods**	ANOVA and t-test used to compare insertion angles.	One-way ANOVA and Pearson correlation applied.	Independent t-tests applied for group comparisons.	Independent t-tests and ANOVA used for statistical analysis.	ANOVA and post-hoc tests used for statistical analysis.
**Results - Participants**	Reports variations in IZC thickness at different angles.	Reports changes in IZC thickness with different angles.	Reports mean IZC thickness differences between skeletal classes.	Reports bone thickness variations at different angles.	Reports variations in IZC thickness by insertion angle.
**Results - Descriptive Data**	Provides mean IZC thickness at different angles (5.2-8.8 mm).	Provides bone thickness measurements at each angle.	Finds Class III patients have significantly greater cortical bone thickness.	Finds bone thickness increases with steeper insertion angles.	Finds best insertion site at 12-17 mm above occlusal plane.
**Results - Outcome Data**	Suggests safe insertion sites at 14-16 mm above occlusal plane and 55°-70° angles.	Finds optimal insertion site at 70° in Durg population.	Suggests optimal insertion zones for Class I and Class III patients.	Identifies safe insertion sites between 16-18 mm from occlusal plane.	Suggests optimal screw size for Dravidian population.

**Results - Main Results**	IZC thickness increases with larger insertion angles.	IZC thickness increases with steeper insertion angles.	IZC thickness varies with skeletal class, affecting insertion site choice.	Compares bone thickness variations by gender.	IZC thickness increases with steeper insertion angles.
**Results - Other Analyses**	Compares right and left side thickness; no significant differences found.	Compares bone thickness variations by gender and insertion angles.	Compares buccal cortical bone thickness and IZC thickness.	Recommends optimal insertion angles for safety.	Compares right and left side measurements.
**Discussion - Key Results**	Finds optimal insertion range but notes limited generalizability due to small sample.	Suggests optimal placement for stability and safety.	Identifies optimal insertion heights and angles.	Acknowledges population-specific limitations.	Recommends ideal insertion heights and angles.
**Discussion - Limitations**	Interprets findings in clinical context of orthodontic miniscrew placement.	Recognizes limitations in sample size and regional specificity.	Acknowledges small sample and need for validation.	Results discussed within the context of Pakistani population.	Recognizes limitations in sample size and demographic scope.
**Discussion - Interpretation**	Results applicable but require validation in larger, diverse populations.	Results interpreted in relation to orthodontic anchorage.	Results linked to IZC miniscrew stability in different skeletal patterns.	Findings not necessarily applicable to other populations.	Results interpreted in relation to Dravidian population.
**Discussion - Generalizability**	Limited sample size but strong methodological approach.	Findings applicable to Indian population but require broader validation.	Results specific to study population; further studies needed.	Considerable sample among reviewed studies, strong statistical approach.	Findings applicable to Dravidian subjects but require broader validation.
**Other Information - Funding**	No external funding reported.	No external funding reported.	No external funding reported.	No external funding reported.	No external funding reported.
**Overall Compliance with STROBE**	Moderate	High	High	High	Moderate

Comments	Moderate compliance, but a larger sample size would be better.	Strong CBCT analysis but limited generalizability.	Important study for skeletal class comparisons.	Relevant study for different angulartions.	Small sample size limits conclusions.

STROBE Checklist for cross-sectional studies (4)

STROBE Item	Lima et al. (2022)	Pan et al. (2024)
**Title & Abstract**	Clearly states study objective and key findings on IZC safe sites for miniscrew insertion.	Clearly states study objective and findings on IZC bone thickness in adolescents at different molar eruption stages.
**Background/Rationale**	Justifies need to evaluate IZC bone thickness variations across	Justifies need to assess IZC bone thickness changes as

	different facial types.	molars erupt to optimize miniscrew placement.
**Objectives**	Determines IZC bone thickness variations in hyperdivergent, neutral, and hypodivergent patients.	Examines IZC bone thickness in adolescents at three eruption stages of the maxillary second molar.
**Study Design**	Retrospective CBCT study.	Retrospective CBCT study.
**Setting**	Conducted at University of São Paulo, Brazil.	Conducted at Zhongshan Hospital, Fudan University, China.
**Participants**	86 CBCT scans (24 hyperdivergent, 30 neutral, 32 hypodivergent).	110 adolescent CBCT scans (44 in early eruption, 30 in mid-eruption, 36 in late eruption).
**Variables**	IZC thickness measured at different heights and insertion angles.	IZC bone thickness measured at 13, 15, and 17 mm above a reference plane.
**Data Sources & Measurement**	CBCT images analyzed using Dolphin Imaging Software.	CBCT images analyzed using SIDEXIS 4 Imaging Software.
**Bias**	Bias minimized by standardizing head positioning and measurement techniques.	Bias minimized through standard imaging protocols and intra-observer calibration.

**Study Size**	Sample size (n=86) determined based on statistical power analysis.	Sample size (n=110) determined using statistical calculations.
**Quantitative Variables**	Bone thickness measured at multiple points between premolars and molars.	Bone thickness measured at mesiobuccal and distobuccal root sites.
**Statistical Methods**	ANOVA and Bonferroni correction applied for multiple comparisons.	Kruskal-Wallis test, Bonferroni correction, and Wilcoxon signed-rank test applied.
**Results - Participants**	Reports distribution of bone thickness by facial type.	Reports variations in IZC thickness across eruption stages.
**Results - Descriptive Data**	Finds optimal IZC insertion zones at 11 mm from alveolar crest for all facial types.	Finds that later eruption stages are associated with thinner IZC bone.
**Results - Outcome Data**	Identifies differences in IZC thickness among facial types.	IZC bone thickness is highest at HB13 and decreases with height.
**Results - Main Results**	Hyperdivergent patients have thinner IZC bone than hypodivergent patients.	Distobuccal root region shows greater bone thickness than mesiobuccal root.
**Results - Other Analyses**	Compares right and left side bone	Compares bone thickness

	thickness differences.	variations by eruption stage.
**Discussion - Key Results**	Recommends individualized miniscrew placement strategies based on facial type.	Recommends earlier miniscrew placement for better stability.
**Discussion - Limitations**	Acknowledges limitations due to sample size and population specificity.	Notes limitations in generalizability to non-adolescent populations.
**Discussion – Interpretation**	Results discussed within the context of skeletal anchorage in orthodontics.	Findings discussed in relation to IZC miniscrew insertion challenges.
**Discussion - Generalizability**	Findings specific to Brazilian population; broader validation required.	Results applicable to adolescents but require validation in adults.
**Other Information - Funding**	No external funding reported.	Funded by the Natural Science Foundation of Fujian Province (2022J011424).
**Overall Compliance with STROBE**	High	High
**Comments**	Strong statistical analysis but requires	Important study on IZC changes

	validation in different populations.	in adolescent development.
